# Piplartine-Inspired 3,4,5-Trimethoxycinnamates: Trypanocidal, Mechanism of Action, and In Silico Evaluation

**DOI:** 10.3390/molecules28114512

**Published:** 2023-06-02

**Authors:** Carlos S. M. B. Filho, Ramon R. P. P. B. de Menezes, Emanuel P. Magalhães, Yunierkis P. Castillo, Alice M. C. Martins, Damião P. de Sousa

**Affiliations:** 1 Department of Pharmaceutical Sciences, Federal University of Paraíba, João Pessoa 58051-900, PB, Brazil; carlosmaia1996@gmail.com; 2 Department of Clinical and Toxicological Analysis, Federal University of Ceará, Fortaleza 60020-181, CE, Brazil; ramonppessoa@ufc.br (R.R.P.P.B.d.M.); emanuelpmagalhaes@gmail.com (E.P.M.); martinsalice@gmail.com (A.M.C.M.); 3 Escuela de Ciencias Físicas y Matemáticas, Universidad de Las Américas, Quito 170125, Ecuador; yunierkis@gmail.com

**Keywords:** natural products, phenylpropanoid, piplartine, alkamide, neglected diseases, molecular docking, *Trypanosoma cruzi*, antiparasitic activity, piperlongumine

## Abstract

Chagas disease (CD) is one of the main neglected tropical diseases that promote relevant socioeconomic impacts in several countries. The therapeutic options for the treatment of CD are limited, and parasite resistance has been reported. Piplartine is a phenylpropanoid imide that has diverse biological activities, including trypanocidal action. Thus, the objective of the present work was to prepare a collection of thirteen esters analogous to piplartine (**1**–**13**) and evaluate their trypanocidal activity against *Trypanosoma cruzi.* Of the tested analogues, compound **11** ((*E*)-furan-2-ylmethyl 3-(3,4,5-trimethoxyphenyl)acrylate) showed good activity with IC_50_ values = 28.21 ± 5.34 μM and 47.02 ± 8.70 μM, against the epimastigote and trypomastigote forms, respectively. In addition, it showed a high rate of selectivity to the parasite. The trypanocidal mechanism of action occurs through the induction of oxidative stress and mitochondrial damage. In addition, scanning electron microscopy showed the formation of pores and leakage of cytoplasmic content. Molecular docking indicated that **11** probably produces a trypanocidal effect through a multi-target mechanism, including affinity with proteins CRK1, MPK13, GSK3B, AKR, UCE-1, and UCE-2, which are important for the survival of the parasite. Therefore, the results suggest chemical characteristics that can serve for the development of new trypanocidal prototypes for researching drugs against Chagas disease.

## 1. Introduction

Chagas disease (CD) is an anthropozoonosis caused by *Trypanosoma cruzi* that has arthropods of the subfamily Triatominae as vectors [1]. Currently, according to the World Health Organization, CD is considered one of the main neglected tropical diseases that promotes relevant socioeconomic impacts in several countries, mainly in Latin America [2]. Data from the Pan American Health Organization (PAHO) estimate that 70 million people in Latin America are at risk of infection with *T. cruzi* and that 6–7 million individuals are infected, causing 14,000 deaths annually as a result of the complications of the disease [3].

The therapeutic options for the treatment of Chagas disease are limited, and there are no new drugs for the treatment since the treatment consists of the use of drugs developed more than 50 years ago (benznidazole and nifurtimox), which are more effective in the acute phase of the disease [4]. Furthermore, these drugs are highly toxic, and the use of nifurtimox has been suspended in some countries, and parasite resistance to these drugs has been reported [5,6]. Thus, the search for new drugs with low toxicity and preferably with new pharmacological targets becomes relevant [7,8].

**Figure 1:**
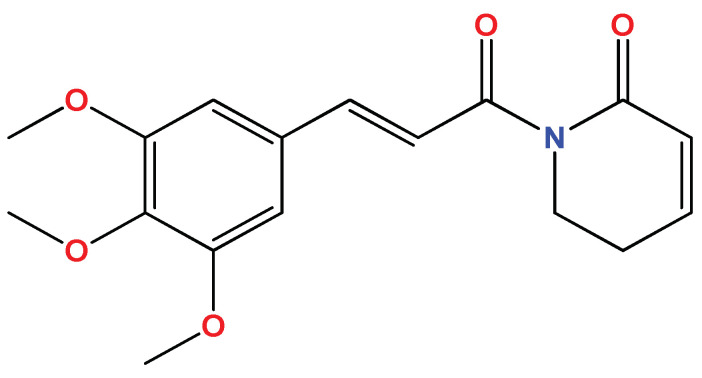
Chemical structure of piplartine.

Natural bioactive compounds are important sources of drug candidates [9]. Piplartine, also called piperlongumine (Figure 1), is a phenylpropanoid in the form of an alkamide typically found in plants of the genus *Piper* and that has diverse biological activities, including cytotoxic, antitumor, schistoicidal, leishmanicidal, and trypanocidal actions [10,11,12,13,14,15,16].

Cell viability studies revealed that piplartine showed greater inhibition of the growth of epimastigote forms of *T. cruzi* (IC_50_ = 21.0 μM) than benznidazole (42.7 μM) [17]. In addition, piplartine reduces the expression of antioxidant enzymes involved in the defense of the parasite, such as tryparedoxine reductase and methionine sulfoxide reductase, which is a possible mechanism of action [16]. Furthermore, esters derived from cinnamic acid derivatives, the precursor of piplartine, showed trypanocidal activity against the epimastigote and trypomastigote forms of *T. cruzi*, especially the compound pentyl *p*-coumarate, which presented IC_50_ of 5.16 and 61.63 µM against the epimastigote and trypomastigote forms, respectively [18]. Considering the trypanocidal potential of piplartine and the fact that there are few data about the trypanocidal action of its derivatives, the objective of this work was to prepare a collection of compounds analogous to piplartine for the evaluation of trypanocidal activity.

## 2. Results and Discussion 

### 2.1. Preparation of Esters ***1**–**13***

**Scheme 1:**
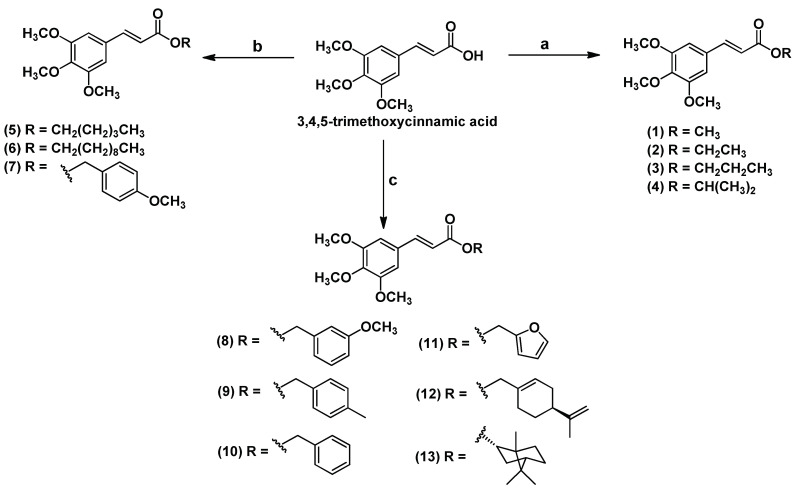
Preparation of esters analogous to piplartine (**1**–**13**). (a) ROH, H_2_SO_4_, reflux; (b) alkyl or aryl halide, acetone, Et_3_N, reflux; (c) ROH, DCC, DMAP, CH_2_Cl_2_.

In this study, thirteen esters derived from 3,4,5-trimethoxycinnamic acid were prepared by maintaining the 3,4,5-trimethoxycinnamate substructure and differing the R moiety with alkyl and aryl substituents. These esters were obtained in a single step and through three methodologies: Fischer esterification [19] for esters: **1** (*R* = methyl), **2** (*R* = ethyl), **3** (*R* = propyl), and **4** (*R* = isopropyl), which showed yields from 58.8–91.1%; esterification with alkyl and aryl halides [11]: **5** (*R* = pentyl), **6** (*R* = decyl), and **7** (*R* = 4-methoxybenzyl) with a yield of 38.0–47.5%; and Steglich esterification [20]: **8** (*R* = 3-methoxybenzyl), **9** (*R* = 4-methylbenzyl), **10** (*R* = benzyl), **11** (*R* = furfuryl), **12** (*R* = (−)-perillyl) and **13** (*R* = (−)-bornyl) with yields ranging from 26.7–62.8%. Scheme 1 illustrates these reactions.

All compounds were characterized by infrared spectroscopy (IR) and nuclear magnetic resonance of hydrogen (^1^H NMR) and carbon thirteen (^13^C NMR). Based on the ^1^HNMR spectrum, the compounds showed an approximate purity greater than 95%. In addition, compounds **8**, **9**, and **12** are unpublished in the literature, and analysis was performed using high resolution mass spectrometry (HRMS). The results and references used in the synthesis of the compounds already described in the literature are available in the Supplementary Materials.

### 2.2. Trypanocidal Evaluation of Esters against T. cruzi

Initially, the prepared esters were submitted to in vitro tests using the microdilution technique in 96-well plates to evaluate the trypanocidal activity against the epimastigote evolutionary form at different concentrations (200–3.12 μg/mL). Then, the compounds with the best activity were tested against the trypomastigote form of *T. cruzi*. In both assays, IC_50_ values were calculated; a compound is considered to have trypanocidal activity when IC_50_ <40 μM [21]. The results are expressed in Table 1.

In addition, in vitro toxicity tests were performed on renal epithelial cells obtained from monkey LLC-MK2 (*Rhesus monkey* kidney) using different concentrations of the compounds (200–25 μg/mL), and the selectivity index (SI) was calculated using the ratio between the CC_50_ (concentration capable of promoting cytotoxicity in 50% of the LLC-MK2 host cells) and the IC_50_. The compounds were considered selective for the parasite when SI ≥ 10 [21]. The results are expressed in Table 2.

Compound **1** presented an IC_50_ of 124.9 ± 36.6 μM against the epimastigote form of the parasite, and comparing it with the analogue with **2** (ethyl radical), it is noted that there was a decrease in potency (321.83 ± 72.89 μM) in contrast to the results obtained by Lima et al., in which ethyl caffeate had an IC_50_ of 18.27 μM against the amastigote form of the parasite [22]. The presence of phenolic hydroxyls in this derivative may have contributed to the better antiparasitic profile compared to **1**, in addition to possible differences in the methodological approach of the biological test.

In relation to the analogues **3** (propyl group) and **4** (isopropyl group), which presented IC_50_ of 321.42 ± 105.95 μM and 265.77 ± 59.22 μM, respectively, comparing these compounds with **1,** it is noticed that the increase of the carbonic side chain by up to three carbons drastically reduces the trypanocidal activity of 3,4,5-trimethoxycinnamates. In addition, the use of the decyl group (**6**) suppressed the trypanocidal activity. 

We hypothesize that the decyl group makes the compound highly lipophilic. Consequently, it can remain trapped in the biological membrane and insoluble in the aqueous medium. To further test this hypothesis, the logarithm of the partition coefficient (logP) was predicted for the 13 compounds evaluated in our research with RDKit (RDKit: Open-source cheminformatics. Version: 2023_03_1; RDKit, 2023. http://www.rdkit.org (accessed on 23 May 2023)). The results of the logP predictions are provided as Table S1 in Supplementary Materials and show that compound **6** is the most lipophilic one. In fact, compound **6** is the only one exceeding a logP value of 5.0, which is the limit set by Lipinski’s rule of five for drug-likeness. The atomic contributions to logP were also explored for each compound using RDKit. The results of the later analysis are depicted in Figure S43 of the Supplementary Material. These predictions reveal that the contribution of the 3,4,5-trimethoxycinnamate ester scaffold to logP is conserved among compounds. On the other hand, the aggregated contribution of longer alkyl substituents is related to larger lipophilic contributions, disfavoring the drug-likeness of compound **6** and making it less soluble and more susceptible to getting trapped in biological membranes [23].

However, the increase in the carbon chain with the introduction of the pentyl radical (**5**) promoted an increase in the antiparasitic action (IC_50_ = 65.50 ± 7.13 μM) in relation to **1**. Similar results were obtained for pentyl *p*-coumarate (IC_50_ of 5.16 μM) against the epimastigote form of *T. cruzi*, compared to methyl *p*-coumarate (IC_50_ = 601 μM), as found by Lopes et al. (2019) [18]. Thus, the replacement of the trimethoxylated aromatic ring by a hydroxylated aromatic ring potentiates the trypanocidal activity. 

Compound **10** showed an IC_50_ of 121.66 ± 42.73 μM against the epimastigote form, and when comparing it with substituted compounds **7** (4-methoxybenzyl group), **8** (3-methoxybenzyl group), and **9** (4-methylbenzyl group) (IC_50_ = 157.37 ± 38.50 μM, 112.36 ± 11.72 μM and 180.49 ± 30.73 μM, respectively), it is noted that there were no significant differences in bioactivity against the epimastigote form of the parasite. However, against the trypomastigote form, the absence of substituents in the aromatic ring resulted in good trypanocidal activity since compound **10** exhibited an IC_50_ of 40.75 ± 12.36 μM and showed selectivity for the parasite (SI = 13.4).

Compound **11**, which has a furfuryl radical, showed the best biological activity against the epimastigote form (IC_50_ = 28.21 ± 5.34 μM) and trypanocidal activity similar to piplartine (21.0 μM). It is worth noting that both substances have an electronegative atom as a heteroatom in a cyclic system, which can contribute to the potentiation of bioactivity. In this study, the reference drug, benznidazole, had an IC_50_ of 42.7 μM [17]. Furthermore, **11** showed a high selectivity index (SI > 22.2), and it was not possible to accurately determine the CC_50_ as the compound did not show toxicity at the highest concentration tested. In addition, compared to trypomastigote cells, the IC_50_ was 47.02 ± 8.70 μM, with no significant difference compared to **10**, also presenting a similar SI. Therefore, further tests were performed with compound **11** to investigate the possible mechanism of trypanocidal activity. Additionally, regarding the investigations of the solubility, stability, and pharmacokinetics of compound **11**, there are no studies available that have addressed these aspects. However, due to its being in the chemical class of esters, it is expected to undergo rapid hydrolysis into 3,4,5-trimethoxycinnamic acid and furfuryl alcohol by plasma esterases [24,25]. Furthermore, previous studies conducted with 3,4,5-trimethoxycinnamic acid in rat plasma have indicated that it has rapid oral absorption and high bioavailability [26]. In general, the collection of compounds has a low polarity, so they should cross biological membranes easily due to their lipophilicity.

### 2.3. Analysis of Cytoplasmic Reactive Oxygen Species (ROS)

**Figure 2:**
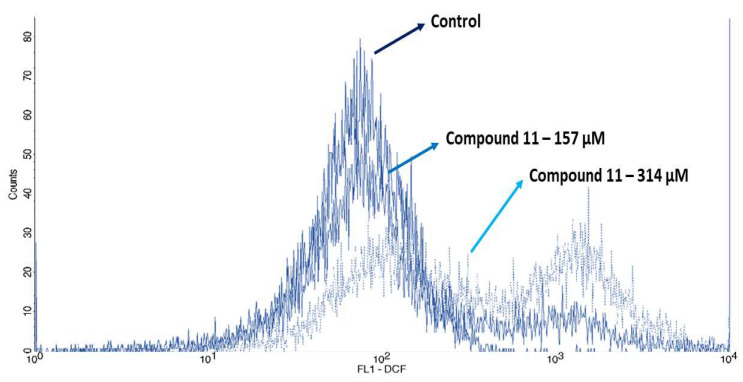
Histogram of staining intensity with DCFH-DA. Experimental groups: control—dark gray; **11** 157 μM—light gray; **11** 314 µM—pale gray.

**Figure 3:**
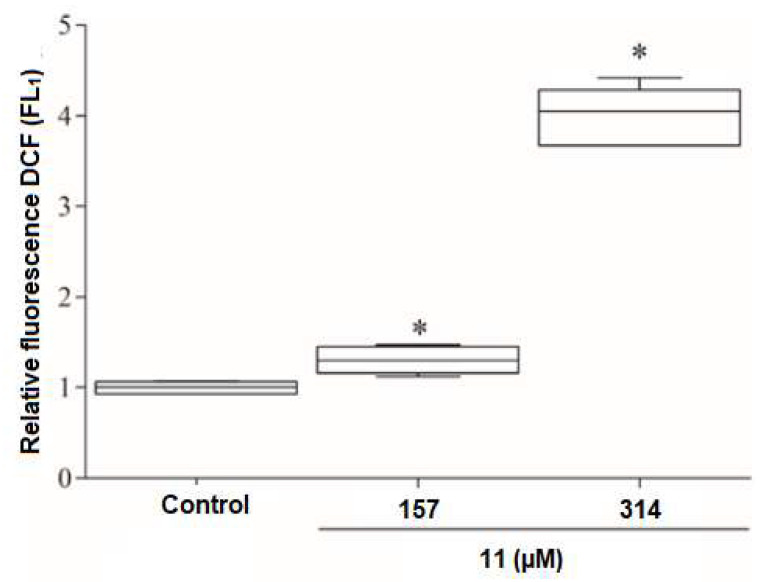
Effect of **11** on the production of cytoplasmic reactive species. * *p* < 0.05 in relation to the control group.

To elucidate the mechanism of action of **11**, the increase in the concentration of ROS inside epimastigote cells of *T. cruzi* was evaluated through fluorescent techniques using 2′,7′–dichlorofluorescein diacetate (DCFH-DA) [27]. The results are illustrated in Figure 2 and Figure 3. 

From these results, it was observed that there was an increase in relative fluorescence in both treated groups when compared to the control (CT). The result indicates that **11** induces oxidative stress in the parasite through increased levels of ROS, similar to piplartine, which has a trypanocidal effect through the induction of oxidative stress [16].

### 2.4. Evaluation of Mitochondrial Transmembrane Potential

**Figure 4:**
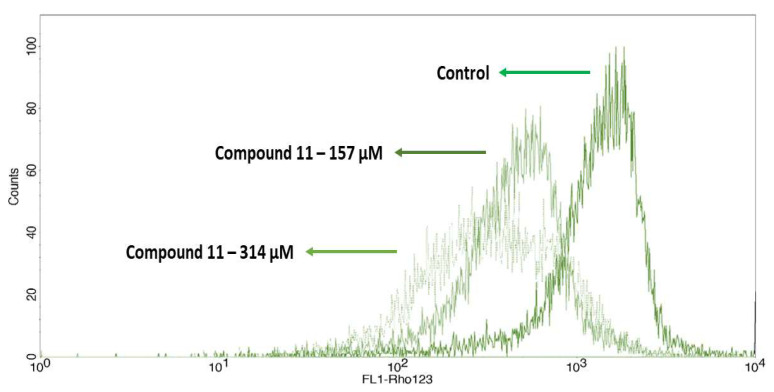
Histogram of staining intensity with Rho 123. Experimental groups: control—dark green; **11** 157 µM—light green; **11** 314 µM—pale green.

**Figure 5:**
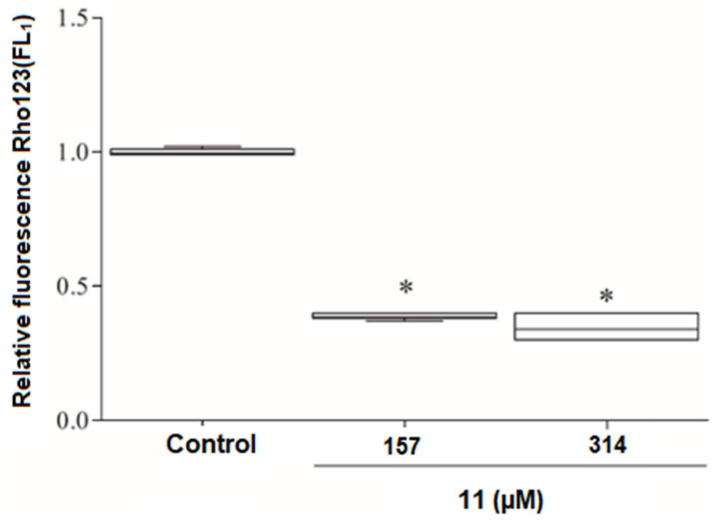
Effect of **11** on mitochondrial transmembrane potential in *T. cruzi* epimastigotes. * *p* < 0.05 in relation to the control group.

In addition, the mitochondrial damage exerted by **11** on the parasite was evaluated through the mitochondrial transmembrane potential assay using the fluorescent dye rhodamine 123 (Rho 123) [22,23]. The results are shown in Figure 4 and Figure 5.

It is concluded that **11** promotes a reduction in the accumulation of rhodamine in the intermembrane space of the mitochondria, indicating mitochondrial injury [28,29]. These data explain the possible trypanocidal mechanisms promoted by **11**. Similar results were obtained through the treatment of the epimastigotes with pentyl *p*-coumarate, both cinnamic derivatives [18].

### 2.5. Structural Evaluation of Epimastigote Forms of T. cruzi Treated with ***11***

**Figure 6:**
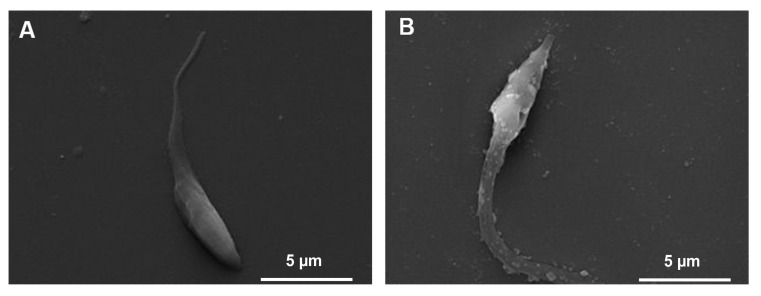
Scanning electron microscopy images of *T. cruzi* epimastigote forms. (**A**) Untreated epimastigotes (control), (**B**) epimastigotes treated with of **11** (157 µM).

In addition, scanning electron microscopy was used to analyze possible morphological alterations of epimastigote forms treated with compound **11**. After 24 h of treatment, ultrastructural alterations were observed in the treated groups in relation to the control, such as pore formation and content leakage in the cytoplasm (Figure 6). Similar results were obtained by treating epimastigote forms with pentyl *p*-coumarate [18].

### 2.6. Analysis of the Cell Death Profile of T. cruzi

Epimastigote forms were used to analyze the cell death profile through labeling with AxPE and 7-AAD. Initially, the cells were divided into four cell populations: viable cells—with a low level of labeling (7AAD−/AxPE−); necrotic cells—labeled with 7-AAD (7AAD+/AxPE−); apoptotic cells—labeled with annexin V-PE (7AAD−/AxPE+); and cells in late apoptosis—doubly labeled (7AAD+/AxPE+).

**Figure 7:**
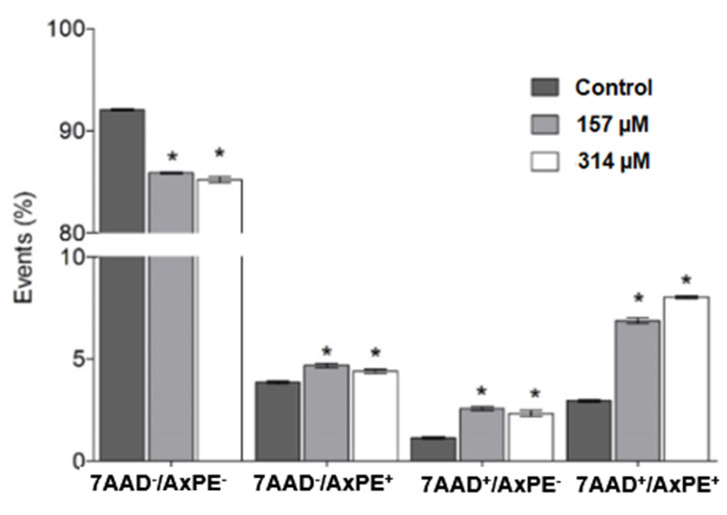
Evaluation of the cell death profile of epimastigote cells treated with **11**. Data were expressed as mean ± SEM and evaluated by two-way ANOVA with Dunnet’s post-test. * *p* < 0.05 in relation to the control group.

The cells were treated with **11** for 24 h at concentrations of 157 and 314 µM. According to the results (Figure 7), there was a reduction in the percentage of viable cells, an increase in the percentage of necrotic cells, an increase in apoptotic cells, and mainly an increase in cells in late apoptosis. 

### 2.7. Molecular Modeling

Modeling studies were performed with the objective of identifying the most probable molecular targets of compound **11** in *T. cruzi*. In brief, potential targets for the compound were first identified through computational target fishing predictions. Next, the compound was docked to the proteins retrieved in the previous step, and binding hypotheses for all of them were generated. Finally, the free energies of binding for the docking-predicted complexes were estimated from molecular dynamics (MD) simulations. It must be stressed that the ranking of the most probable targets of compound **11** was performed based on the MD-predicted binding energies. In addition to providing more accurate results than molecular docking in terms of binding energies, free energies calculated from MD trajectories avoid the biases associated with comparing docking scores across different targets.

The objective of computational target-fishing methods is to identify potential targets for chemical compounds. They rely on the similarity principle and use databases containing confirmed ligand-receptor interactions as references. Unfortunately, these databases are biased against human targets. For this reason, the homology-based approach described in the methods section was implemented to identify potential *T. cruzi* targets for **11**. The list of potential molecular targets obtained following this procedure is presented in Table 3. Among the proteins listed in Table 3, TUB-A and TUB-B form a heterodimer that contains the inhibitors’ binding site at the interface of both subunits [30]. Thus, the tubulin (TUB) heterodimer was assembled, taking as reference the crystal structure of the tubulin from Rattus norvegicus in complex with an inhibitor (PDB code 6H9B). **11** was then docked into the dimer interface ligand binding pocket of TUB. In addition, it has been described that the ubiquitin-conjugating enzyme E2 can be inhibited by directly blocking the interaction of ubiquitin with the catalytic cysteine residue as well as by binding to an allosteric site located distant from the catalytic site [31]. For this reason, the binding of **11** to both the catalytic and the allosteric sites of the ubiquitin-conjugating enzyme E2 was explored.

Molecular docking of **11** of the 19 targets listed in Table 3 was performed as described in the Methods section, and its results are summarized in Table 4. In 13 out of the 21 studied systems, more than one probable binding mode is predicted for **11**, leading to 42 ligand-receptor complexes for further analyses. According to the obtained results, the best docking scores are observed for the AKR, MetAP2, SIR2, and MPK13 proteins, while the worst-scored targets are MPK4, PK-2, and UCE-1. Despite these differences in scores across targets, the visual inspection of the 42 complexes reveals interactions that could stabilize all these complexes and that, in all cases, the ligand occupies the receptors’ binding sites. Overall, this data is not enough to prioritize a subset of the **11** potential targets studied. 

We have previously shown that the additional study of ligand-receptor complexes through MD simulations for the estimation of their free energy of binding can aid the prioritization of the potential molecular targets of chemical compounds [15,18]. Taking this into account, MD simulations and MM-PBSA calculations were performed for the 42 predicted ligand-receptor complexes as described in the methods section, and their results are presented in Table 5.

**Figure 8:**
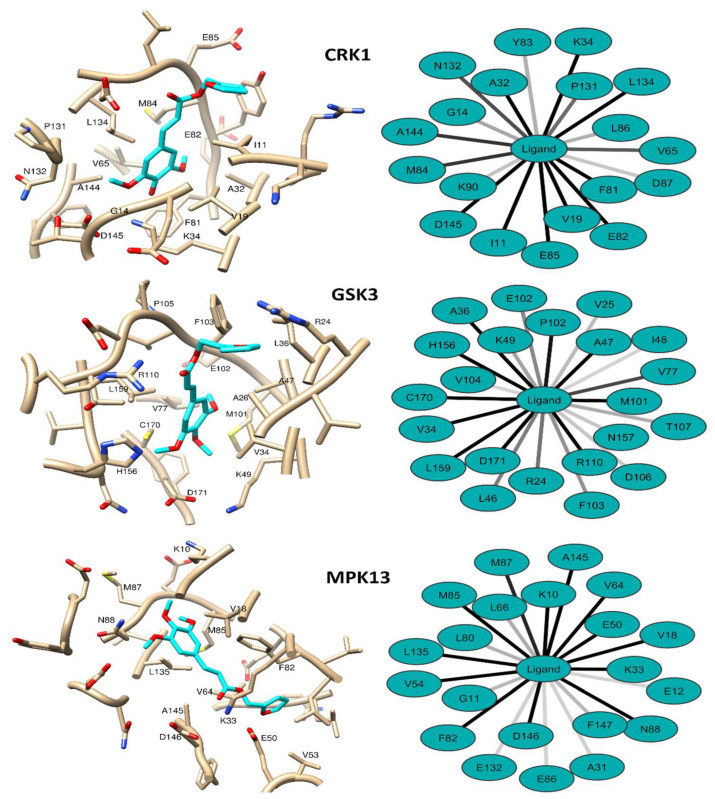
Predicted binding poses of **11** to the protein kinases CRK1 (**top**), GSK3 (**center**), and MPK13 (**bottom**) and networks of interactions between the ligand and the receptors. The interaction networks were constructed from the 200 MD snapshots used for MM-PBSA calculations. Darker lines indicate more frequent interactions. Receptors are represented in gray and the ligand in cyan, while non-carbon atoms use the following coloring scheme: red for oxygen, blue for nitrogen, and yellow for sulfur. Only residues interacting with the receptors in more than 50% of the analyzed MD snapshots are labeled.

The obtained results show that for 7 targets plus the catalytic domain of UCE-1, the predicted complexes are unstable (ΔG > 0). Interestingly, there are 12 targets yielding negative estimations of the free energy of binding that can be interpreted as feasible **11**-receptor complexes. Among them, the PGFS, TOP2, LGL, MetAP2, SIR2, PPIase, and the catalytic site of UCE-2 present ΔG values higher than −3 kcal/mole, indicating low stability of the complexes predicted for them. On the other hand, better free energies of binding are predicted for AKR, CRK1, MPK13, GSK3B, and the allosteric sites of UCE-1 and UCE-2, being the ubiquitin-conjugating enzymes E2 and those with the lowest predicted ΔG. To get more insights into the possible mechanism of action of **11**, the predicted complexes with the later listed targets were analyzed more in detail. CRK1, GSK3B, and MPK13 are protein kinases sharing the same overall folding and containing one ATP binding site. The predicted binding poses of **11** to these protein kinase receptors as well as the network of interactions that it forms with the receptors are presented in Figure 8. Figures representing molecular structures were prepared with UCSF Chimera [32], and interaction networks were produced with Cytoscape [33].

All in all, protein kinase receptor **11** occupies the ATP binding sites. In CRK1 and GSK3, the ligand orients its trimethoxyphenyl moiety toward the bottom of the binding pockets, while the furan substituent occupies the entrance of the binding site, orienting toward the solvent. Both complexes are mainly stabilized by hydrophobic and Van der Waals interactions. The predicted complex with GSK3 shows the possibility of hydrogen bonds between the ligand and the side chains of R110 and C170 through the compound’s carbonyl group and one of the methoxy substituents of the phenyl ring, respectively. Similarly, a hydrogen bond is predicted between one of the methoxy groups of the ligand and K34 of CRK1 in 97% of the MD snapshots used for MM-PBSA calculations.

In the case of MPK13, **11** is predicted to bind in an orientation rotated 180° relative to that observed in GSK3 and CRK1. That is, the furan substituent occupies the bottom of the binding site, while the trimethoxyphenyl moiety is located at the mouth of the pocket and exposed to the solvent. As for GSK3 and CRK1, the ligand is mainly stabilized through hydrophobic and Van der Waals interactions. The predicted complex is further stabilized by hydrogen bonds between the carboxyl group of the compound and the side chains of K33 and D146 and between one of the methoxy substituents and the side chain of N88. The slightly lower free energy of binding estimated for MPK13 relative to GSK3 and CRK1 could be explained by the larger frequency of hydrogen bond formation observed in the first. 

**Figure 9:**
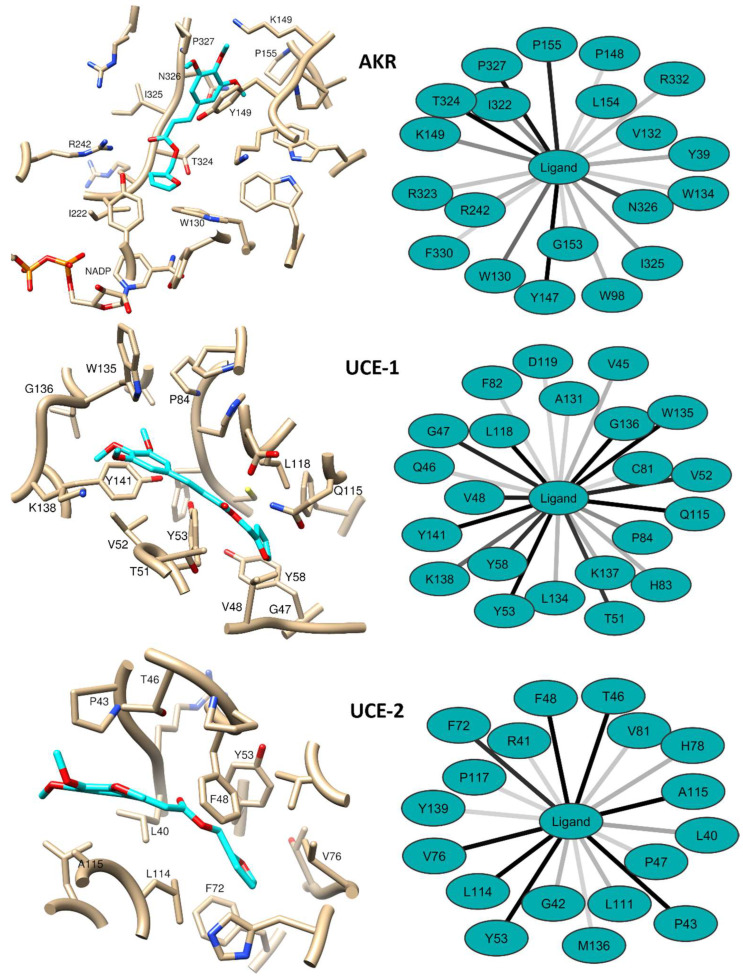
Predicted binding poses of **11** to AKR (**top**) and the allosteric binding sites of UCE-1 (**center**) and UCE-2 (**bottom**). The networks of interactions between the ligand and the receptors are also represented on the left. The interaction networks were constructed from the 200 MD snapshots used for MM-PBSA calculations. Darker lines indicate more frequent interactions. Receptors are represented in gray and the ligand in cyan, while non-carbon atoms use the following coloring scheme: red for oxygen, blue for nitrogen, orange for phosphorus, and yellow for sulfur. Only residues interacting with the receptors in more than 50% of the analyzed MD snapshots are labeled.

The predicted binding poses of **11** to the predicted non-protein kinase receptors are presented in Figure 9. The predicted complex of **11** with AKR shows that the ligand does not directly interact with the NADP cofactor. Instead, it binds at the tunnel that provides access to the cofactor, potentially preventing the enzyme’s substrates access to NADP and their reduction. In this complex, the ligand positions itself favorably to form hydrogen bonds with either the side chain of R242 or the backbone of I325 through its carboxyl oxygen. Additional stability is provided to this complex by the stacking of the furan ring and the trimethoxyphenyl substituent in front of Y39 and Y147, respectively.

According to the predicted free energies of binding, the most stable binding of **11** is obtained at the allosteric sites of UCE-1 and UCE-2. Although these two ubiquitin-conjugating enzymes E2 share only 34% identity, the predicted binding poses of the chemical to them are highly similar. In both cases, the furan group positions itself toward the interior of the cavity, while the trimethoxyphenyl moiety is located at the entrance of the binding pocket. The furan ring is located in both receptors in regions populated with aromatic residues, which favors its stacking with them. Furthermore, the presence of the bulkier part of the ligand (the trimethoxyphenyl group) at the mouth of the site gives stability to the distorted conformations of the enzymes required for the inhibitory effect on ubiquitination previously observed in one human homolog protein [31].

There are several residues at these receptors that could hydrogen bond the ligand; however, this type of interaction is only observed between the ligand’s carboxyl oxygen and either the backbone of V48 or the side chain of Q115 of UCE-1. In contrast, no hydrogen bond is predicted between the ligand and UCE-2. The lack of hydrogen bonds in the UCE-2 complex, despite the highly similar predicted binding modes of **11** to both targets, can be explained by the V48P and Q115L mutations present in UCE-2. This result agrees with the only hydrogen bond observed in the X-ray complex of an allosteric inhibitor with the human Cdc34 ubiquitin-conjugating enzyme E2 [31]. As previously reported in the aforementioned study, the complexes herein reported with UCE-1 and UCE-2 are mainly stabilized by hydrophobic and Van der Waals interactions.

Considering all the obtained results, we postulate that **11** might produce its trypanocidal effect through a multi-target mechanism interfering with different cellular processes. This hypothesis is further supported by the fact that one of the more probable targets of the compound predicted in this research has been shown to be effective for trypanosome inhibition. For example, CRK1, MPK13, and GSK3B are protein kinases that share less than 30% identity between them and play different roles in *T. cruzi*. GSK3B is essential for the life cycle of *T. brucei,* and its inhibition leads to a trypanocidal effect [34]. Specifically, indirubin derivatives have demonstrated both GSK3B inhibition and antiparasitic activity against *T. brucei* and *T. cruzi* [35,36]. The second protein kinase identified as a potential target of **11** is CRK1. This protein is involved in the cell cycle regulation of trypanosomes, specifically in the G_1_/S transition, and in protein transport [37,38,39]. The last protein kinase identified as a potential target of the studied compound is MPK13, which is a member of the mitogen-activated protein kinase family. Although no specific role has been reported for MPK13 in *T. cruzi*, several studies point to a central role for this family of proteins in the regulation and development of the parasite [40,41,42,43].

Among the other probable targets of **11**, AKR is annotated as a putative aldo-keto reductase protein in the UniProt database. This superfamily of proteins is related to the maintenance of the redox balance in the cell through the reduction of a variety of substrates [44]. Finally, the ubiquitin conjugating enzymes E2 have been demonstrated to be essential for trypanosome survival [45]. The results obtained by Rojas et al. in [45] also show that the ubiquitin conjugating enzyme E2 from *T. brucei* is inhibited by an allosteric human enzyme inhibitor. This last result supports the use of the analog allosteric binding site in *T. cruzi* for UCE-1 and UCE-2 inhibition.

Previous studies on the trypanocidal activity of piplartine have shown that it induces the expression of proteins related to the control of oxidative stress in *T. cruzi* [16]. On the other hand, several studies demonstrate that interference with the ubiquitination pathway in human cells leads to oxidative stress [46,47]. More importantly, it was reported that the antitumoral activity of piplartine is due to its interference with the ubiquitination pathway, which causes oxidative stress in tumoral cells [48,49]. These precedents and our modeling results strongly point to the ubiquitin-conjugating enzymes E2 of *T. cruzi* here studied as the preferred targets of the assayed piplartine derivatives. 

The presented computational models can guide new experiments to fully clarify the trypanocidal mode of action of this series of chemical compounds and optimize their bioactivity. Our results allow for narrowing the space of molecular targets to be experimentally evaluated as well as prioritizing the proteins with the highest probabilities of serving as targets for these compounds. Future experimental efforts will be directed toward the evaluation of the predicted inhibitory activity of compound **11** against its most probable targets. These assays will include enzymatic inhibition experiments. Afterwards, site-directed mutagenesis experiments can be designed to validate the predicted binding mode of the compound to its confirmed targets. The results of such experiments will enable new rounds of structure-based drug design focusing on the improvement of the potency of piplartine derivatives as trypanocidal agents.

## 3. Materials and Methods

### 3.1. Chemical Characterization of Compounds

The infrared was performed by FTIR spectrophotometry, Cary 630 FTIR Agilent Technologies, potassium bromide pills, and frequency measurement in cm^−1^. The 1H and 13C NMR spectra were obtained on a Mercury-Varian machine operating at 400 MHz and 100 MHz and a Varian-NMR-System machine operating at 500 and 125 MHz. CDCl3 was used as the deuterated solvent. High-resolution mass spectra were performed on Ultraflex II TOF/TOF equipment with a high-performance solid-state laser (λ = 355 nm) and a reflector, using the MALDI technique. Samples were loaded on a steel plate (MTP 384 steel base; Bruker Daltonics Gmbh, Bremen, Germany). Finally, for the analysis of the melting point of the compounds, the heating plate of a device from Microquímica Equipamentos LTDA, model MQAPF 302, was used.

### 3.2. Preparation of ***1**–**4***

In a 50 mL flat-bottomed flask, 3,4,5-trimethoxycinnamic acid (0.1 g; 0.42 mmol) and 20 mL of the respective alcohol (methanol, ethanol, *n*-propanol, or isopropyl alcohol) were added. Then, 0.2 mL of concentrated H_2_SO_4_ was slowly added. Thus, the reaction was carried out under reflux and magnetic stirring for 6 to 24 h. All reaction steps were monitored by thin-layer chromatography (TLC). After completion of the reaction, the alcohol was partially removed under reduced pressure, then the product was transferred to a separatory funnel with 10 mL of distilled water and extracted using 3 × 10 mL of ethyl acetate. The organic phase was treated with 5% sodium bicarbonate (3 × 10 mL), washed with 10 mL of distilled water, and dried with anhydrous sodium sulfate. The solvent was evaporated under reduced pressure. For the purification of **4**, it was necessary to perform adsorption chromatography on a silica gel 60 column, using as the mobile phase a mixture of hexane and ethyl acetate with an increasing polarity gradient [19].

### 3.3. Preparation of ***5**–**7***

In a 50 mL flat-bottomed flask, 0.1 g (0.42 mmol) of 3,4,5-trimethoxycinnamic acid, 0.43 mmol of the corresponding halide (1-bromopentane, 1-bromodecane, or 4-methoxybenzyl chloride), 5 mL of anhydrous acetone, and 1.68 mmol of triethylamine were added. The reaction was carried out under reflux and magnetic stirring for 24 h and monitored using TLC. The acetone was removed under reduced pressure, then the reaction product was transferred to a separatory funnel and extracted with 10 mL of distilled water and dichloromethane (3 × 10 mL). The organic phase was treated with 5% sodium bicarbonate (3 × 10 mL), 10 mL of distilled water, dried with anhydrous sodium sulfate, and filtered. After extraction, the solvent was evaporated under reduced pressure, and the products were purified by column adsorption chromatography on silica gel 60 using a mixture of hexane and ethyl acetate with an increasing polarity gradient as the mobile phase [11,44].

### 3.4. Preparation of ***8**–**13***

The 3,4,5-trimethoxycinnamic acid (0.1 g; 0.42 mmol), 0.54 mmol of the respective alcohols (3-methoxybenzyl alcohol, 4-methylbenzyl alcohol, benzyl alcohol, furfuryl alcohol, (−)-perillyl alcohol, or (−)-borneol), 0.015 g of 4-dimethylaminopyridine (DMAP), and 6.3 mL of dichloromethane were added to a 50 mL flat bottom flask. After 5 min 0.11 g (0.54 mmol) of *N*,*N*′-dicyclohexylcarbodiimide (DCC) was added, and the reaction was carried out under magnetic stirring and at room temperature for 24–48 h. The solvent was partially removed under reduced pressure, and the reaction product was filtered three times to remove the 1,3-dicyclohexyl urea (DCU). The filtrate was transferred to a separatory funnel and extracted with 10 mL of distilled water and dichloromethane (3 × 10 mL). The organic phase was treated with 10 mL of 5% sodium bicarbonate, 10 mL of distilled water, and dried with anhydrous sodium sulfate. The reaction product was purified on a silica gel 60 column using a mixture of hexane and ethyl acetate [45,46,50].

### 3.5. In Vitro Cytotoxicity Assay in LLC-MK2 Cells

For the cytotoxicity analysis, the (3-[4,5-dimethylthiazol-2-yl]-2,5 diphenyl tetrazolium bromide) (MTT) assay was used, in which the LLC-MK2 cells were cultured in Dulbecco’s Modified Eagle Medium (DMEM), then exposed to the piplartine analogues at different concentrations (25–200 μg/mL) and incubated for 24 h at 37 °C. Then MTT was added, and the cells were incubated for 4 h, after which 10% sodium dodecyl sulfate was added. Cell viability measurements were performed in a microplate reader (Biochrom^®^ Asys Expert Plus, Cambridge, UK) at a wavelength of 570 nm, and the CC_50_ was defined. To calculate the relative cell viability, cells treated only with sterile phosphate-buffered saline were used as a negative control. The experiments were conducted in triplicates [51].

### 3.6. Evaluation of In Vitro Trypanocidal Activity in Y Strains of T. cruzis

#### 3.6.1. Test on Epimastigote Forms of *T. cruzi*

Initially, epimastigotes were cultured and distributed in 96-well plates at a density of 10^6^ cells/mL in liver infusion tryptose medium supplemented with 10% fetal bovine serum (FBS) and 1% antibiotic. Then, the cells were treated with piplartine analogues at different concentrations and incubated at 28 °C for 24 h. To quantify the growth inhibition of the parasites, a Neubauer chamber was used, and the IC_50_ was defined [52]. 

#### 3.6.2. Assay on Trypomastigote Forms of *T. cruzi*

Trypomastigote forms were obtained by infecting LLC-MK2 cells with the parasite; in this way, the cells were incubated for 24 h in T-25/75 cm^2^ flasks at 37 °C in an atmosphere with 5% CO_2_ in DMEM medium supplemented with 2% FBS and 1% antibiotic. Subsequently, the trypomastigote forms were distributed in 96-well plates treated with piplartine analogues at different concentrations (200; 100; 50; 25; 12.5; 6.25; 3.12 μg/mL) and incubated at 37 °C for 24 h. The assessment of cell viability was performed by counting, and the IC_50_ was defined [53]. 

#### 3.6.3. The Trypanocidal Mechanism of Action Assays

Thirteen compounds were tested against the epimastigotes form of *T. cruzi.* The compound that presented a lower IC_50_ of epimastigotes compared to the other compounds and benznidazole, the standard drug, was chosen to investigate the trypanocidal mechanism of action.

##### Analysis of Reactive Cytoplasmic Oxygen Species

The DCFH-DA assay was used to evaluate the increase in ROS concentration in the cytoplasm of epimastigote cells treated with **11**. Initially, 10^6^ epimastigote cells/mL were added to 24-well plates in the presence of **11** (157 μM and 314 µM). After 3 h, 10 µL of a solution of DCFH-DA (20 µM in DMSO) was added. After 24 h, the cells were centrifuged (2800 RPM for 7 min), washed, and resuspended in 500 µL of phosphate buffered saline for flow cytometer analysis. Thus, the level of labeling in each group was analyzed by the geometric mean of the fluorescence intensity [54], and the results were expressed as relative fluorescence intensity according to the following formula (Equation (1)):

(1) $$\mathrm{Relative}\;\mathrm{fluorescence}=\frac{\mathrm{mTest}}{\mathrm{mControl}}$$

mTEST = geometric mean of the interest group; mCONTROL = geometric mean of the control group.

#### 3.6.4. Evaluation of Mitochondrial Transmembrane Potential

Initially, **11** (157 and 314 µM) and epimastigote forms (10^6^ cells/mL) were added in 24-well plates. After 24 h, the cells were centrifuged and washed with phosphate-buffered saline. Subsequently, the cells were resuspended in 100 µL of phosphate-buffered saline, and 5 µL of Rho123 solution (10 µg/mL) was added. Thus, the samples were incubated in the dark for 30 min, washed, and resuspended with phosphate-buffered saline (500 µL/tube) for analysis by flow cytometry [55]. Results were evaluated by relative fluorescence intensity, calculated as described above.

#### 3.6.5. 7-AAD Assay

The epimastigote forms of *T. cruzi* were cultivated at a density of 10^6^ cells/mL in the presence of **11** (157 and 314 µM) for 24 h in a stove. Then, the cells were transferred to cytometry tubes, centrifuged at 4000 RPM for 5 min and washed twice with phosphate-buffered saline and binding buffer (pH 7.4; a compound of HEPES solution 10 mM; NaCl 140 mM; and CaCl_2_ 2.5 mm). After the last centrifugation, the pellet was resuspended in 100 µL binding buffer, 5 µL 7-AAD (0.5 mg/mL), and 5 µL Ax/PE. The procedure was performed using a commercial kit (Annexin V PE Apoptosis Detection Kit I, BD Biosciences) and following the manufacturer’s guidelines. After 15 min of incubation in the dark, 400 µL of binding buffer was added to each tube, and the cells were analyzed on the FACSCalibur (BD Biosciences). For each tube, a minimum of 10^4^ cells were analyzed to quantify the percentage of unlabeled cells and were either singly or double labeled with 7-AAD and Ax/PE. For this, an argon laser (488 nm) was used to excite the fluorochromes. Ax/PE-labeled cells were read by the FL_2_ detector (563 to 606 nm, yellow fluorescence), while 7-AAD-labeled cells were read at 615 to 645 nm wavelength (red fluorescence, FL_3_ detector).

#### 3.6.6. Evaluation of Morphological Changes in *T. cruzi* Induced by **11**

To evaluate the changes induced by **11** in the ultrastructure of *T. cruzi*, the epimastigote forms were incubated with them for 24 h. After this period, the samples were centrifuged (5000 rpm; 10 min), the supernatant discarded, and the pellet resuspended in glutaraldehyde solution (2.5%) and incubated for 2 h. The fixed samples were again centrifuged and exposed to increasing concentrations of ethanol (30, 50, 70, 90, and 100%) for dehydration, followed by centrifugation (5000 rpm; 5 min). Finally, the cells were transferred to the surface of circular coverslips (15 mm) and dried in a CO_2_ oven. The coverslips were covered with a gold layer (20 nm thick) using the QT150 ES-Quorum Metallizer and analyzed in a Quanta 450 FEG-FEI scanning electron microscope to observe changes in the three-dimensional structure of the epimastigote forms of *T. cruzi*.

### 3.7. Statistical Analysis

For statistical analysis, the GraphPad Prism 5 program (GraphPad Software, San Diego, CA, USA) was used. Values were expressed as mean ± mean standard error (S.P.M), and data were calculated using one-way analysis of variance (ANOVA), followed by Dunnett’s post-test. Significance was defined as * *p* < 0.05.

### 3.8. Molecular Docking: Targets Selection

Computational target fishing was performed for **11** using the Similarity Ensemble Approach (SEA) [56]. The identified potential targets were used as input to a NCBI Blast (Altschul, 1997) search against the *T. cruzi* proteome (taxid: 5693) on the Reference proteins (refseq_protein) database. From the Blast results, *T. cruzi* proteins sharing at least 35% sequence identity with the SEA predicted potential targets and with alignment coverage higher than or equal to 70% were selected as potential targets of **11**. To avoid duplications in the *T. cruzi* list of potential targets for **11**, it was manually curated, and duplicate sequences (e.g., the same protein with different UniProt Accession Codes) were removed.

OpenEye’s Omega [57] and Molcharge [58] software were employed to generate the most stable three-dimensional conformation of **11** and to add atomic partial charges to it, respectively. The X-ray structure of PGFS, PDB code 4GIE, was retrieved from the Protein Data Bank [59]. The rest of the potential targets of the compound had no solved structure, and homology models were generated for them using the SWISS-MODEL server [60]. If necessary, catalytically important cofactors, including metal ions, were added to the homology models, taking their positioning in the models’ templates as a reference.

Molecular docking was performed following the same consensus ranking approach as in our previous publications [15,18]. The Gold [61] software was selected for molecular docking. The receptor binding sites were defined as any residue within 6 Å from the ligands co-crystallized with the homology model templates or with *T. cruzi* PGFS. The CHEMPLP scoring function was selected for primary docking and was set up to explore 30 different possible binding modes of the ligand to each receptor. The search efficiency parameter of Gold was set to 200%, and the 30 possible binding modes obtained for each receptor were rescored with the GoldScore, ChemScore, and ASP scoring functions of Gold. 

The selection of the most probable binding modes of **11** to every receptor was made using the same consensus scoring strategy described in our previous publications [15,18]. For this, the scores predicted for binding pose *I* according to each scoring function *j* (*S_i_*_,*j*_) are aggregated according to Equation (2).

(2) $$Z_{i}=\frac{1}{4}\sum_{j}\frac{S_{i,j}-\bar{S_{j}}}{std\left(S_{j}\right)}$$

where $\bar{S_{j}}$ and $std\left(S_{j}\right)$ represent the average and standard deviation of scoring function *j*, respectively, across the 30 predicted binding poses of **11**. Either any predicted binding pose with an aggregated score of *Z_i_* > 1 or the highest-scored conformation of the ligand were considered for further calculations.

### 3.9. Molecular Dynamics Simulations and MM-PBSA Calculations

MD simulations and MM-PBSA calculations took place with Amber 18 [62]. All systems were subject to the same simulation procedure that included energy minimization, heating, equilibration, MD production runs, and free energy of binding estimation from MD snapshots. The MD simulations and MM-PBSA calculations protocol were the same as those employed in our previous publication [18].

In brief, the ff14SB force field was used to parametrize proteins, while the gaff force field was employed for non-amino acid molecules. The complexes were embedded in truncated octahedron boxes and solvated with TIP3P water molecules. Excess charges were neutralized by the addition of either Na^+^ or Cl^−^ counterions.

The solvated and neutralized systems were minimized in two stages, the first of which consisted of 500 steps of the steepest descent method followed by 500 cycles of conjugate gradient with all atoms except solvent constrained with a force constant of 500 kcal/mol·Å^2^. The second stage of energy minimization sequentially implemented 500 steps of the steepest descent algorithm and 1000 cycles of conjugate gradient. No constraint was applied during the second minimization stage. During both energy minimizations, long-range electrostatic interactions were treated with the PME method using a cutoff distance of 10 Å.

Next, the systems were heated from 0 K to 300 K at constant volume along 10,000 steps with a time step of 2 fs. The PME cutoff for heating was set to 12 Å and the solute was constrained with a force constant of 10 kcal/mol·Å^2^. The temperature was controlled using a Langevin thermostat with a collision frequency of 1.0 ps^−1^. The SHAKE algorithm was employed to constrain the bonds involving hydrogen atoms, and their interactions were omitted during heating and all subsequent MD steps.

Afterward, the solvated complexes were equilibrated for 100 ps using a time step of 2 fs at a constant pressure of 1 bar and 300 K constant temperature. The temperature was controlled during heating and pressure with isotropic position scaling, setting a relaxation time of 2 ps. The PME cutoff for equilibration was set to 12 Å. Then, 20 different production runs were performed for each equilibrated complex. Different random initial atomic velocities were set for each of these production runs. The parameters for the production runs were the same as those used during the equilibration stage.

MM-PBSA calculations were employed to estimate the free energy of binding **11** to its potential receptors. These were performed with the MMPBSA.py script provided by Amber 18 [63]. For each system, MM-PBSA calculations were performed over 200 MD snapshots (one every 200 ps) selected from the 20 production runs with the ionic strength set to 100 mM.

## 4. Conclusions 

In the present study, it was possible to prepare thirteen esters analogous to piplartine (**1**–**13**), which are structurally related. In the evaluation of trypanocidal activity against *T. cruzi*, compound **11** showed good activity with IC50 values = 28.21 ± 5.34 μM and 47.02 ± 8.70 μM, against the epimastigote and trypomastigote forms, respectively. In addition, it showed a high rate of selectivity for the parasite (SI > 10). The trypanocidal mechanism of action occurs through the induction of oxidative stress and mitochondrial damage in the parasite’s cells. It was also noted that **11** promotes parasite cell death through necrosis processes and, mainly, late apoptosis. These results could be verified through scanning electron microscopy, which showed that there was a formation of pores and leakage of cytoplasmic content from epimastigote cells. Molecular docking indicated that **11** probably produces a trypanocidal effect through a multi-target mechanism interfering with different cellular processes, including affinity with the protein kinases CRK1, MPK13, and GSK3B, essential for the parasite’s life cycle; the AKR protein involved in the control of cellular oxidative stress; and the ubiquitin E2 conjugating enzymes (UCE-1 and UCE-2), important for the survival of the parasite. Therefore, it is concluded that it was possible to establish chemical characteristics that can serve as a reference for the development of new trypanocidal prototypes with a better biological profile, and other studies can be carried out with piplartine analogous compounds to search for new drugs with trypanocidal activity.

## Figures and Tables

**Table 1:** Trypanocidal activity of compounds **1**–**13** against the Y strain of *T. cruzi*.

		Epimastigote		Trypomastigote	
Ester	Substituent	IC_50_ (μg/mL)	IC_50_ (μM)	IC_50_ (μg/mL)	IC_50_ (μM)
1	Methyl	31.5 ± 9.23	124.9 ± 36.6	-	-
2	Ethyl	85.68 ± 19.41	321.83 ± 72.89	-	-
3	Propyl	90.1 ± 29.7	321.42 ± 105.95	-	-
4	Isopropyl	74.5 ± 16.6	265.77 ±59.22	-	-
5	Pentyl	20.2 ± 2.2	65.50 ±7.13	-	-
6	Decyl	n*	n*	-	-
7	4-methoxybenzyl	56.4 ± 13.8	157.37 ± 38.50	-	-
8	3-methoxybenzyl	40.27 ± 4.2	112.36 ± 11.72	64.44 ± 19.01	179.80 ± 53.04
9	4-methylbenzyl	61.80 ± 10.52	180.49± 30.73	39.92 ± 7.34	123.06 ± 21.44
10	Benzyl	39.99 ± 14.03	121.66 ± 42.73	13.38 ± 4.06	40.75 ± 12.36
11	Furfuryl	8.98 ± 1.70	28.21 ± 5.34	14.97 ± 2.77	47.02 ± 8.70
12	(−)-Perillyl	70.08 ± 25.56	188.16 ± 68.63	38.72 ± 5.03	103.96 ± 13.51
13 Positive control	(−)-Bornyl Benznidazole	155.1 ± 43.8 23.0 ± 3.3	414.19 ± 116.97 115.1 ± 16.3	- 32.3 ± 6.4	- 161.4 ± 31.8

n* = could not determine IC_50._ Benznidazole was used as a positive control.

**Table 2:** Cytotoxicity of compounds **1**–**13** against the LLC-MK2 cells.

		LLCK-MK2		Selectivity Index (SI)	
Ester	Substituent	CC_50_ (μg/mL)	CC_50_ (μM)	Epimastigote	Trypomastigote
1	Methyl	126.4 ± 25.4	501.1 ± 100.7	4.01	-
2	Ethyl	>200.0	>751.1	>2.3	-
3	Propyl	147.5 ± 15.6	526.2 ± 55.6	1.6	-
4	Isopropyl	>200.0	>713.5	>2.7	-
5	Pentyl	>200.0	>648.6	>9.9	-
6	Decyl	>200.0	>528.4	n*	-
7	4-methoxybenzyl	196.2 ± 26.6	547.4 ± 74.2	3.5	-
8	3-methoxybenzyl	100.3 ± 21.7	279.9 ± 60.5	2.5	1.6
9	4-methylbenzyl	182.8 ± 23.7	533.9 ± 69.2	3.0	4.6
10	Benzyl	183.0 ± 47.8	557.3 ± 125.0	4.6	13.7
11	Furfuryl	>200.0	>628.3	>22.2	>13.4
12	(−)-Perillyl	105.9 ± 13.8	284.3 ± 37.1	1.5	2.7
13	(−)-Bornyl	71.2 ± 12.7	190.1 ± 33.9	0.5	-

n* = could not determine CC_50_.

**Table 3:** Potential molecular targets of **11** identified through computational target fishing.

*T. cruzi* Target ^(a)^	Description	ID
PGFS_TRYCC	9,11-endoperoxide prostaglandin H2 reductase (Prostaglandin F2-alpha synthase)	PGFS
Q4D831_TRYCC	Aldo/keto reductase	AKR
Q4CRI4_TRYCC	Cell division protein kinase 2 (Cell division protein kinase 2)	CRK1
Q4CPK2_TRYCC	Cell division related protein kinase 2	CRK3
Q4DF38_TRYCC	Cystathionine beta-synthase	CBS
Q4CST7_TRYCC	Cysteine synthase	CS
Q4DE53_TRYCC	DNA topoisomerase 2	TOP2
Q4D7B4_TRYCC	Lactoylglutathione lyase-like protein	LGL
Q4DRC6_TRYCC	Methionine aminopeptidase 2 (MAP 2, Peptidase M)	MetAP2
Q4D3A0_TRYCC	Mitogen-activated protein kinase	MPK4 ^(c)^
Q4CNV0_TRYCC	NAD-dependent protein deacylase (Regulatory protein SIR2 homolog 5)	SIR2
Q4DQ47_TRYCC	Peptidylprolyl isomerase	PPIase
Q4E4I5_TRYCC	Protein kinase	MPK13 ^(c)^
Q4D8B2_TRYCC	Protein kinase	PK-2
Q4DJM4_TRYCC	Protein kinase	PK-3
Q4E1X0_TRYCC	Protein kinase	GSK3B
Q4CLA1_TRYCC	Tubulin alpha chain	TUB-A ^(b)^
Q4DQP2_TRYCC	Tubulin beta chain	TUB-B ^(b)^
Q4DI08_TRYCC	Ubiquitin-conjugating enzyme E2	UCE2-1
Q4DDU2_TRYCC	Ubiquitin-conjugating enzyme E2	UCE2-2

^(a)^ UniProt entry name. ^(b)^ The TUB-A and TUB-B heterodimer is identified as TUB from here on. ^(c)^ IDs inferred from homology with Leishmania homolog proteins.

**Table 4:** Results of the docking of **11** to its potential targets.

Target	Conformer	CHEMPLP		GoldScore		ChemScore		ASP		Consensus Z-Score
		Score	Z-Score	Score	Z-Score	Score	Z-Score	Score	Z-Score	
PGFS	1	55.38	0.87	19.87	0.18	24.93	1.95	39.55	1.34	1.08
	2	56.13	1.11	24.85	0.20	24.17	1.58	39.52	1.33	1.05
AKR	1	53.63	2.08	36.75	2.69	25.77	2.25	36.00	1.25	2.07
	2	51.84	1.62	30.31	1.21	21.83	0.82	37.50	1.51	1.29
	3	53.64	2.08	28.11	0.70	21.56	0.73	36.48	1.33	1.21
	4	51.27	1.48	29.07	0.92	23.33	1.37	32.02	0.55	1.08
CRK1	1	49.44	1.46	24.23	1.02	19.09	0.47	26.28	1.93	1.22
	2	50.32	1.90	26.12	1.56	19.37	0.64	23.26	0.53	1.16
	3	49.03	1.25	23.59	0.84	19.94	1.00	25.00	1.34	1.11
CRK3	1	57.19	2.91	21.34	0.80	20.57	1.79	31.60	1.65	1.79
	2	48.99	1.10	24.53	1.62	19.11	0.95	31.77	1.70	1.34
	3	55.01	2.43	25.61	1.89	20.19	1.58	31.03	1.51	1.85
CBS	1	50.53	2.99	23.97	1.49	17.83	2.43	31.51	2.08	2.25
	2	46.89	1.64	24.58	1.65	15.36	-0.06	31.29	2.03	1.32
TOP2	1	47.31	1.72	32.39	2.81	16.30	0.54	26.99	2.63	1.93
LGL	1	55.63	0.97	15.12	0.41	27.54	2.03	36.55	1.97	1.35
MetAP2	1	60.84	1.78	30.12	0.69	24.99	0.75	41.40	2.07	1.32
	2	61.59	2.03	19.17	-1.66	29.69	2.56	39.58	1.37	1.08
	3	59.04	1.14	32.47	1.20	24.44	0.54	39.12	1.19	1.02
MPK4	1	50.54	3.00	22.44	0.58	17.34	0.64	21.51	0.56	1.20
SIR2	1	60.77	2.31	30.55	0.92	22.18	0.99	39.46	1.64	1.46
	2	57.06	1.35	29.65	0.74	23.36	1.49	36.44	1.08	1.17
	3	53.41	0.42	34.40	1.68	22.69	1.21	36.83	1.15	1.12
PPIase	1	50.32	0.85	30.10	3.17	22.73	1.27	32.76	0.28	1.39
	2	53.58	2.40	23.44	1.02	22.61	1.18	30.56	-0.39	1.05
MPK13	1	71.06	2.90	26.69	0.70	27.89	1.97	33.87	1.70	1.82
	2	59.72	1.15	26.64	0.69	26.05	1.47	30.02	0.81	1.03
	3	60.42	1.26	28.42	1.06	23.69	0.82	30.46	0.91	1.01
PK-2	1	46.08	2.41	22.62	0.28	17.35	1.40	24.65	1.35	1.36
	2	38.93	0.31	20.27	0.27	19.70	2.29	25.51	1.56	1.11
PK-3	1	46.69	1.22	27.52	1.76	19.56	1.95	29.60	3.30	2.06
	2	48.90	2.13	25.68	1.29	18.73	1.36	23.66	1.11	1.47
GSK3B	1	50.14	2.01	26.51	1.21	18.85	1.86	28.11	2.40	1.87
	2	51.23	2.34	23.63	0.56	18.10	1.45	26.16	1.48	1.46
TUB ^(a)^	1	53.55	1.91	29.68	1.54	19.22	1.72	26.38	0.75	1.48
CS	1	47.31	1.72	32.39	2.81	16.30	0.54	26.99	2.63	1.93
UCE-1 ^(b)^	1	43.01	2.19	23.54	0.57	10.70	0.31	19.28	0.44	0.88
UCE-2 ^(b)^	1	51.21	2.26	27.26	1.36	15.18	1.80	25.84	3.03	2.11
UCE-1 (Allosteric) ^(b)^	1	46.93	1.70	27.96	0.22	17.77	0.73	26.64	0.91	0.89
UCE-2 (Allosteric) ^(b)^	1	60.80	2.36	29.53	2.27	24.61	1.22	31.77	2.16	2.00
	2	57.56	1.48	23.81	0.72	25.68	1.61	28.80	1.26	1.27
	3	56.57	1.21	27.01	1.58	23.44	0.79	27.79	0.96	1.14

^(a)^ Tubulin dimer. ^(b)^ Ubiquitin-conjugating enzymes E2 were modeled considering both the catalytic and allosteric binding sites.

**Table 5:** Predicted free energies of binding of **11** to the studied targets and their components. All energy values are presented in kcal/mole.

Target	Conformer	MM-PBSA Component							ΔG of Binding
		VDWAALS	EEL	EPB	ENPOLAR	EDISPER	ΔG Gas	ΔG Solv	
PGFS	1	−26.81	−8.70	19.75	−20.47	34.45	−35.51	33.73	−1.78
	2	−23.22	−6.78	18.11	−17.95	31.13	−30.00	31.30	1.29
AKR	1	−35.42	−16.38	30.94	−26.84	42.96	−51.81	47.07	−4.74
	2	−35.46	−6.28	21.80	−27.15	44.13	−41.74	38.78	−2.96
	3	−35.89	−14.97	30.91	−27.18	44.40	−50.86	48.13	−2.73
	4	−35.49	−14.97	31.93	−26.99	43.63	−50.46	48.57	−1.89
CRK1	1	−39.02	−12.98	31.65	−28.90	48.23	−52.00	50.98	−1.02
	2	−41.25	−18.48	35.57	−30.00	49.90	−59.73	55.46	−4.26
	3	−38.32	−12.72	32.55	−28.05	47.09	−51.05	51.59	0.55
CRK3	1	−34.41	−13.30	37.46	−25.21	45.51	−47.70	57.75	10.05
	2	−35.85	−11.81	37.11	−26.92	46.36	−47.65	56.55	8.90
	3	−30.46	−6.27	24.34	−21.98	40.79	−36.73	43.16	6.43
CBS	1	−41.85	−14.31	39.71	−29.62	52.03	−56.16	62.12	5.96
	2	−43.60	−18.91	42.39	−29.24	52.38	−62.51	65.53	3.02
TOP2	1	−52.25	−20.66	47.10	−34.06	59.24	−72.91	72.28	−0.63
LGL	1	−34.52	−37.75	50.92	−29.54	48.32	−72.27	69.70	−2.57
MetAP2	1	−35.64	−7.59	30.17	−25.34	45.31	−43.23	50.13	6.90
	2	−41.01	−16.04	39.30	−29.09	51.58	−57.05	61.79	4.74
	3	−41.68	−25.67	43.35	−30.00	53.26	−67.35	66.60	−0.75
MPK4	1	−36.97	−6.80	30.09	−26.60	46.31	−43.77	49.79	6.02
SIR2	1	−36.53	−6.07	21.95	−26.86	45.76	−42.59	40.86	−1.73
	2	−46.74	−8.72	31.61	−32.30	55.14	−55.46	54.45	−1.01
	3	−47.25	−7.09	29.33	−32.61	55.42	−54.33	52.14	−2.20
PPIase	1	−27.05	−4.07	15.06	−21.34	35.67	−31.13	29.40	−1.73
	2	−26.16	−4.95	16.80	−20.33	34.49	−31.11	30.96	−0.15
MPK13	1	−46.26	−23.09	42.13	−33.58	55.34	−69.35	63.90	−5.45
	2	−46.10	−20.12	38.41	−33.00	55.24	−66.22	60.66	−5.57
	3	−45.34	−19.69	38.34	−32.83	55.02	−65.03	60.53	−4.50
PK-2	1	−32.10	−7.09	24.91	−23.10	40.63	−39.19	42.44	3.25
	2	−33.47	−9.74	30.99	−24.02	43.00	−43.21	49.97	6.76
PK-3	1	−42.66	−13.34	37.95	−29.44	50.88	−55.99	59.39	3.40
	2	−38.93	−11.23	39.12	−28.07	49.97	−50.17	61.02	10.85
GSK3B	1	−39.33	−16.59	33.76	−31.25	49.74	−55.93	52.26	−3.67
	2	−36.30	−6.22	25.25	−26.98	45.99	−42.52	44.26	1.74
TUB-B	1	−47.14	−6.78	31.54	−32.26	55.49	−53.92	54.77	0.85
CS	1	−38.85	−21.03	42.10	−28.73	50.21	−59.88	63.58	3.70
UCE-1	1	−30.01	−5.49	21.02	−21.13	36.14	−35.51	36.04	0.53
UCE-2	1	−32.57	−9.21	24.57	−23.05	37.62	−41.78	39.14	−2.64
UCE-1 (Allosteric)	1	−47.88	−3.69	24.61	−32.90	53.44	−51.57	45.16	−6.42
UCE-2 (Allosteric)	1	−41.24	−1.97	15.27	−28.68	45.65	−43.21	32.25	−10.97
	2	−36.78	−3.21	16.38	−27.01	42.67	−39.99	32.03	−7.96
	3	−40.42	−3.17	16.67	−28.61	45.09	−43.59	33.16	−10.43

## Data Availability

The data presented in this study are available in the article and in the Supplementary Materials.

## Supplementary Materials

The following supporting information can be downloaded at: https://www.mdpi.com/article/10.3390/molecules28114512/s1. Chemical characterization compounds **1**–**13**; Atomic contributions to logP for compounds **1**–**13**; Predicted logarithm of the partition coefficients for compounds **1**–**13**. References [11,15,50,64,65,66,67] are cited in supplementary materials.
