# Supplementary material for: Piplartine-Inspired 3,4,5-Trimethoxycinnamates: Trypanocidal, Mechanism of Action, and In Silico Evaluation
Source: Molecules. 2023 Jun 2;28(11):4512. doi: 10.3390/molecules28114512 (PMC10254306; doi:10.3390/molecules28114512)
Supplement: Supplementary file 1 [file molecules-28-04512-s001.zip › molecules-2388849-supplementary.pdf]

Supporting Information

Article

Piplartine-Inspired 3,4,5-Trimethoxycinnamates: Trypanocidal, Mechanism of Action, and In Silico Evaluation

Carlos S. M. B. Filho [1], Ramon R. P. P. B. de Menezes [2], Emanuel P. Magalhães [2], Yunierkis P. Castillo [3], Alice M. C. Martins [2] and Damião P. de Sousa [1,*]

[1] Department of Pharmaceutical Sciences, Federal University of Paraíba, 58051-900 João Pessoa, PB, Brazil; carlosmaia1996@gmail.com

[2] Department of Clinical and Toxicological Analysis, Federal University of Ceará, Fortaleza 60020-181, CE, Brazil; ramonppessoa@ufc.br (R.R.P.P.B.d.M.); martinsalice@gmail.com (E.P.M.); martinsalice@gmail.com (A.M.C.M.)

[3] Escuela de Ciencias Físicas y Matemáticas, Universidad de Las Américas, Quito 170125, Ecuador; yunierkis@gmail.com

* Correspondence: damiao_desousa@yahoo.com.br

Abstract: Chagas disease (CD) is one of the main neglected tropical diseases that promote relevant socioeconomic impacts in several countries. The therapeutic options for the treat-ment of CD are limited, and parasite resistance has been reported. Piplartine is a phe-nylpropanoid imide that has diverse biological activities, including trypanocidal action. Thus, the objective of the present work was to prepare a collection of thirteen esters anal-ogous to piplartine (**1–13**) and evaluated the trypanocidal activity against *Trypanosoma cruzi.* Of the tested analogues, compound **11** ((*E*)-furan-2-ylmethyl 3-(3,4,5-trimethoxy-phenyl)acrylate) showed good activity with IC_{50} values = 28.21±5.34 μM and 47.02±8.70 μM, against the epimastigote and trypomastigote forms, respectively. Also, it showed a high rate of selectivity to the parasite. The trypanocidal mechanism of action occurs through the induction of oxidative stress and mitochondrial damage. In addition, scan-ning electron microscopy showed a formation of pores and leakage of cytoplasmic con-tent. Molecular docking indicated that **11** probably produces a trypanocidal effect through a multi-target mechanism, including affinity with protein CRK1, MPK13, GSK3B, AKR, UCE-1, and UCE-2, important for the survival of the parasite. Therefore, the results suggest chemical characteristics that can serve for the development of new trypanocidal prototypes for researching drugs against Chagas disease.

Keywords: natural products; phenylpropanoid; piplartine; alkamide; neglected diseases; molecular docking; Trypanosoma; cruzi; antiparasitic activity

Experimental:

Chemical characterization compounds **1-13**

(*E*)-Methyl 3-(3,4,5-trimethoxyphenyl)acrylate (**1**): white solid; Yield: 91.1%; Reaction time: 6 h; m.p.: 96-96ºC (lit. 95-98ºC); TLC (7:3 Hex/EtOAc), R_f = 0.55; IR *ν*max (KBr, cm^{-1}): 3006, 2946, 2838, 1697, 1634, 1583, 1505, 1469, 1249, 1128, 818; **^{1}H NMR ($CDCl_3$, 500 MHz):** δH 7.60 (*d*; *J*=15.92 Hz; 1H), 6.74 (*s*; 2H), 6.33 (*d*; *J*=15.92 Hz; 1H), 3.87 (*s*; 9H), 3.79 (*s*, 3H); **^{13}C NMR ($CDCl_3$, 125 MHz):** δC 167.4, 153.5, 144.9, 140.3, 130.0, 117.1, 105.4, 61.1, 56.3, 51.8 [1].

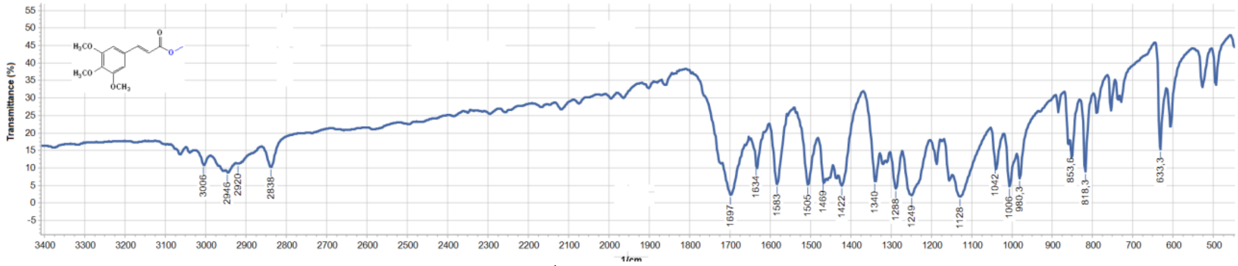

Figure S1: Infrared spectrum (KBr, cm^{-1}) of (*E*)-methyl 3-(3,4,5-trimethoxyphenyl)acrylate (**01**).

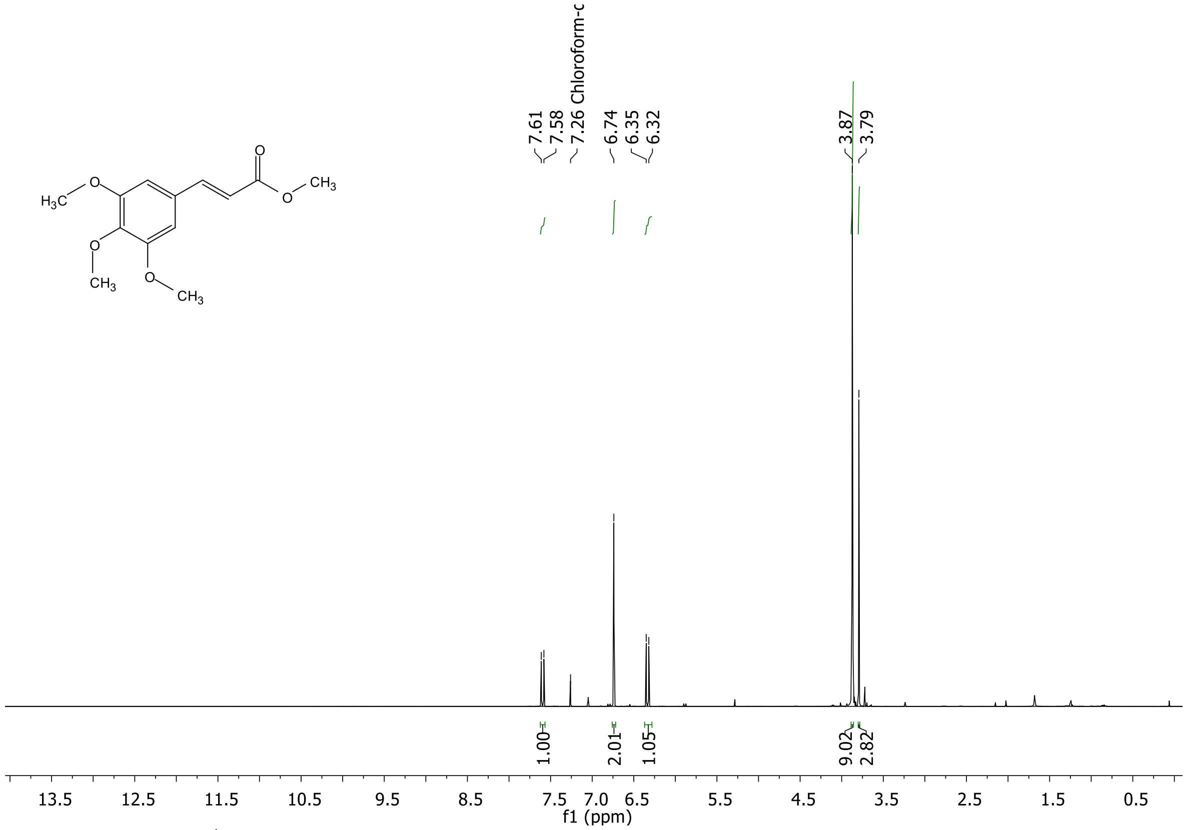

Figure S2: ^{1}H NMR (500MHz, $CDCl_3$) spectrum of (*E*)-methyl 3-(3,4,5-trimethoxyphenyl)acrylate (**01**).

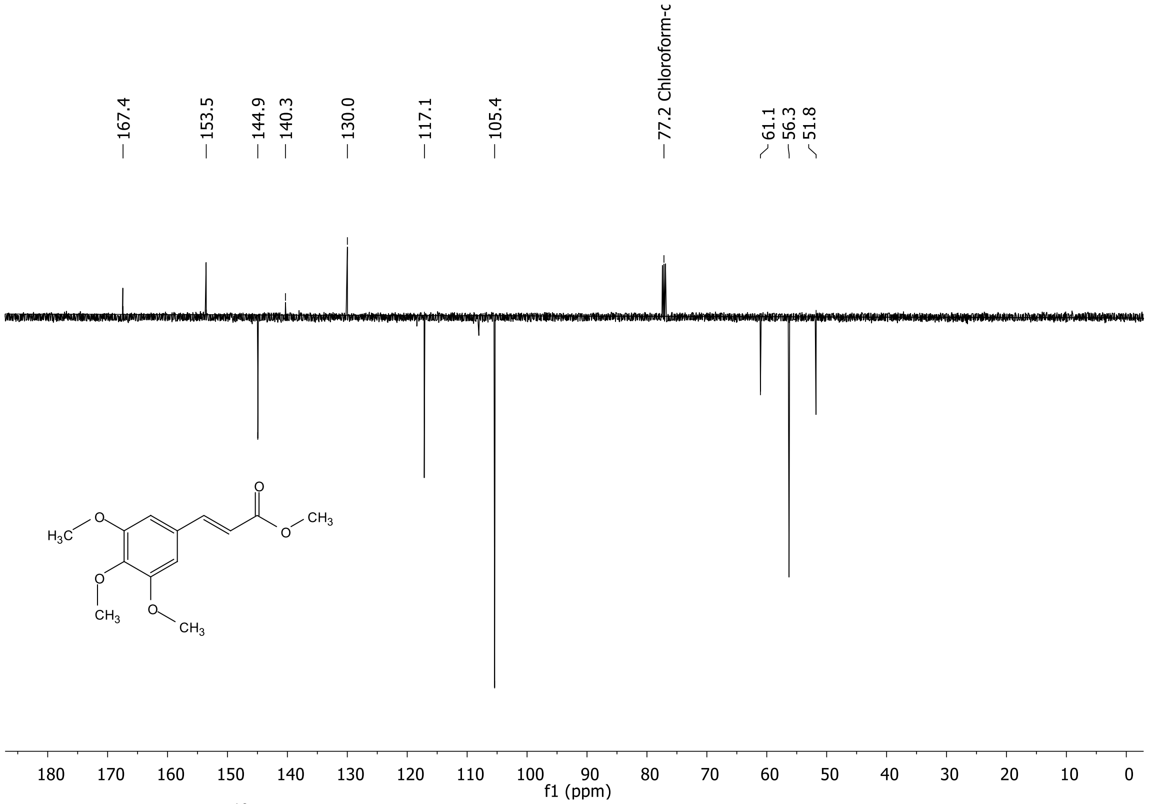

Figure S3: APT-^{13}C NMR (125 MHz, $CDCl_3$) spectrum of (*E*)-methyl 3-(3,4,5-trimethoxyphenyl)acrylate (**01**).

(*E*)-Ethyl 3-(3,4,5-trimethoxyphenyl)acrylate (**2**): white solid; Yield: 82.5%; Reaction time: 6 h; m.p.: 65-66ºC (lit. 68-69ºC); TLC (7:3 Hex/EtOAc), R_f = 0.56; IR *v*max (KBr, cm^{-1}): 3004, 2946, 2838, 1702, 1636, 1582, 1504, 1456, 1280, 1123, 827; **^{1}H NMR ($CDCl_3$, 500 MHz):** δH 7.59 (*d*; *J*=15.91 Hz; 1H), 6.74 (*s*; 2H), 6.34 (*d*; *J*=15.91 Hz; 1H), 4.25 (*q*; *J*= 7.13 Hz; 2H); 3.87 (*s*; 9H); 1.34 (*t*; *J* =7.13 Hz; 3H); **^{13}C NMR ($CDCl_3$, 125 MHz):** δC 167.2, 153.8, 144.8, 140.5, 130.3, 118.0, 105.7, 61.3, 60.9, 56.5, 14.7 [2].

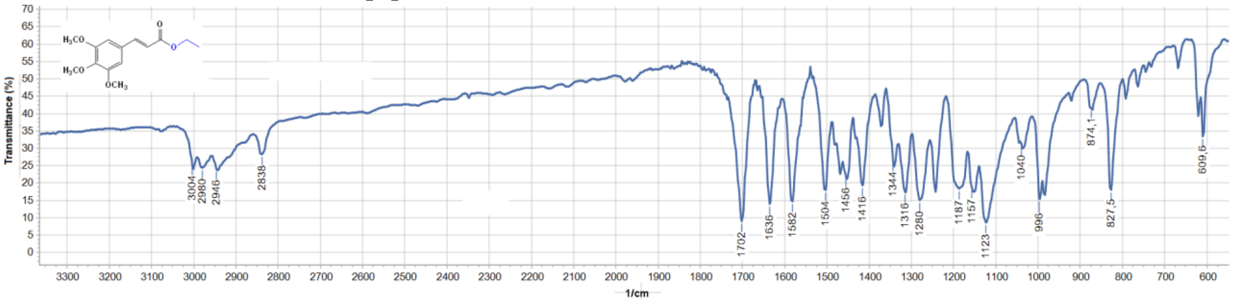

Figure S4: Infrared spectrum (KBr, cm^{-1}) of (*E*)-ethyl 3-(3,4,5-trimethoxyphenyl)acrylate (**2**).

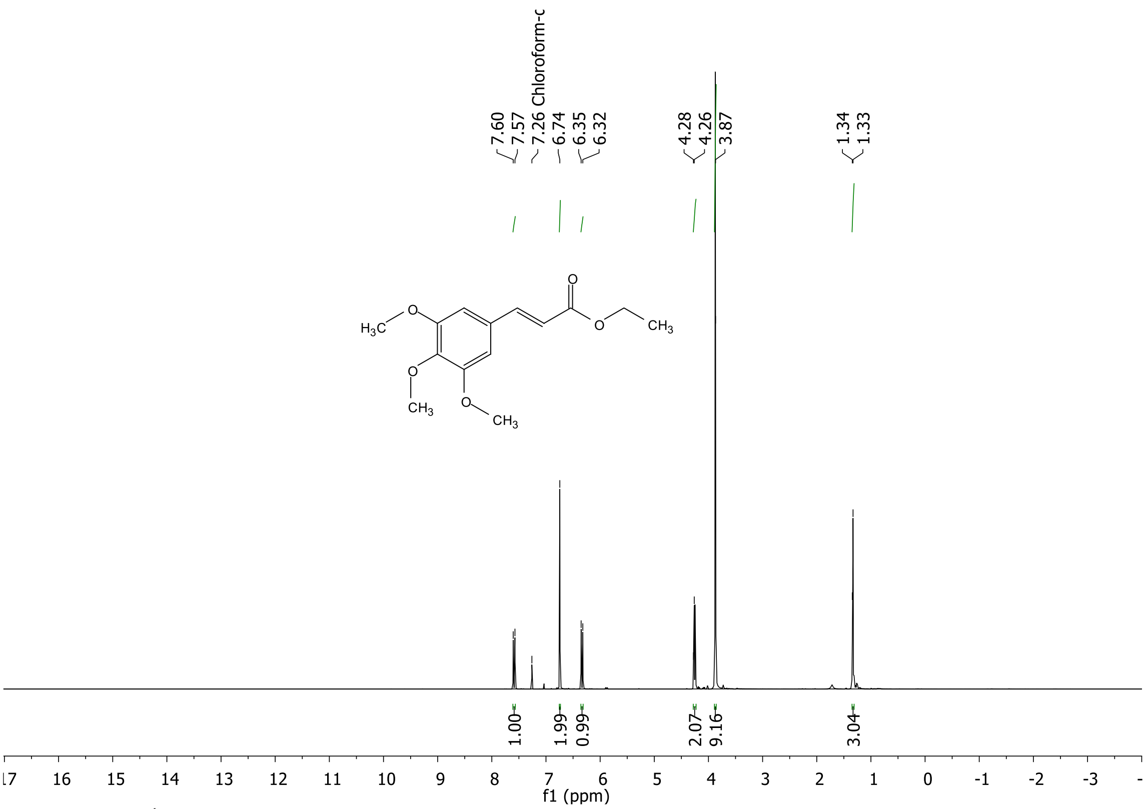

Figure S5: ^{1}H NMR spectrum (500 MHz, $CDCl_3$) of (*E*)-ethyl 3-(3,4,5-trimethoxyphenyl)acrylate (**2**).

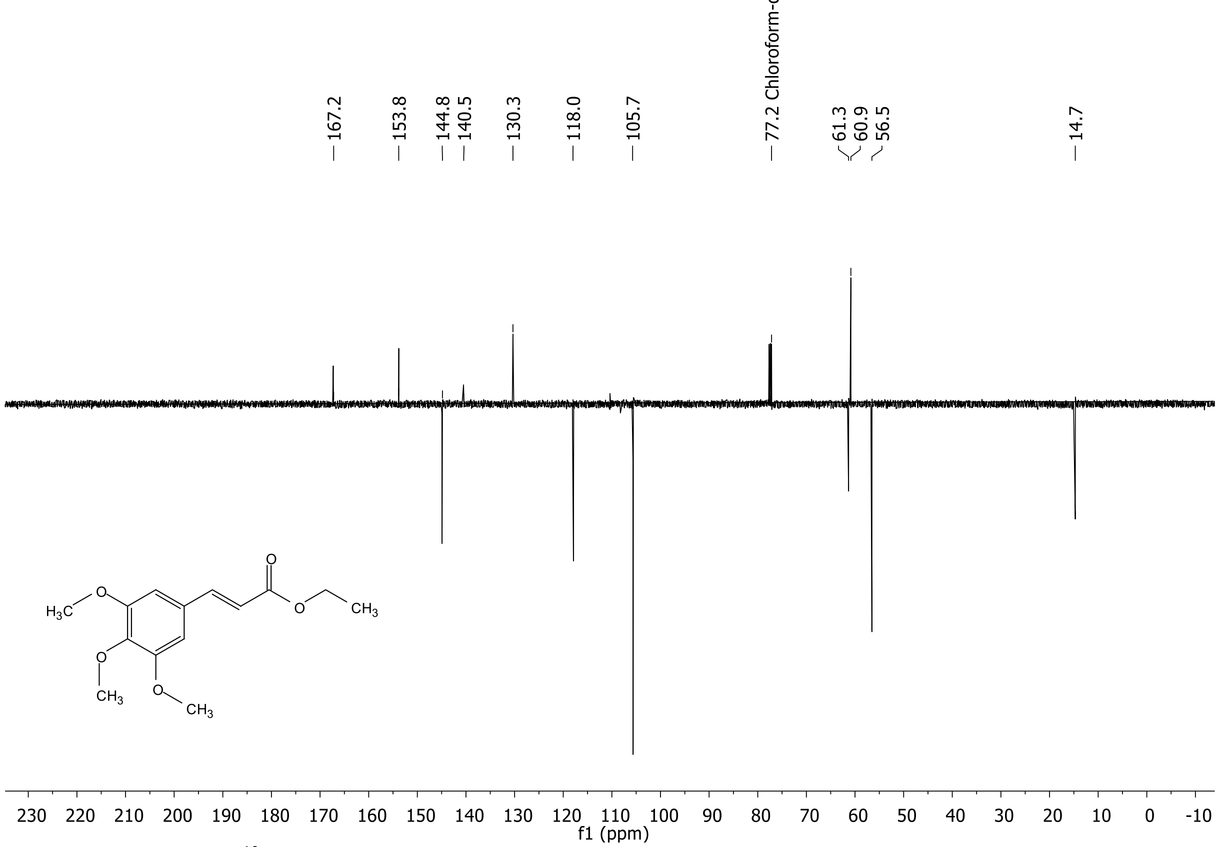

Figure S6: APT-^{13}C NMR spectrum (125 MHz, $CDCl_3$) of (*E*)-ethyl 3-(3,4,5-trimethoxyphenyl)acrylate (**02**).

(*E*)-propyl 3-(3,4,5-trimethoxyphenyl)acrylate (**3**): yellow solid; Yield: 79.9%; Reaction time: 6 h; m.p.: 73-74ºC (lit. 58ºC); TLC (7:3 Hex/EtOAc), R_f = 0.58; IR *v*max (KBr, cm^{-1}): 3001, 2939, 2840, 1707, 1640, 1581, 1505, 1472, 1276, 1126, 846; **^{1}H NMR ($CDCl_3$, 400 MHz):** δH 7.58 (*d*; *J*=15.90 Hz; 1H), 6.74 (*s*; 2H), 6.34 (*d*; *J*=15.90 Hz; 1H), 4.16 (*t*; *J*=6.73 Hz; 2H), 3.87 (*s*; 9H), 1.74-1.67 (*m*; 2H), 0.99 (*t*; *J* = 7.41 Hz; 3H); **^{13}C NMR($CDCl_3$, 100 MHz):** δC 167.2, 153.5, 144.7, 140.2,

130.1, 117.6, 105.3, 66.3, 61.0, 56.2, 22.2, 10.6 [1]

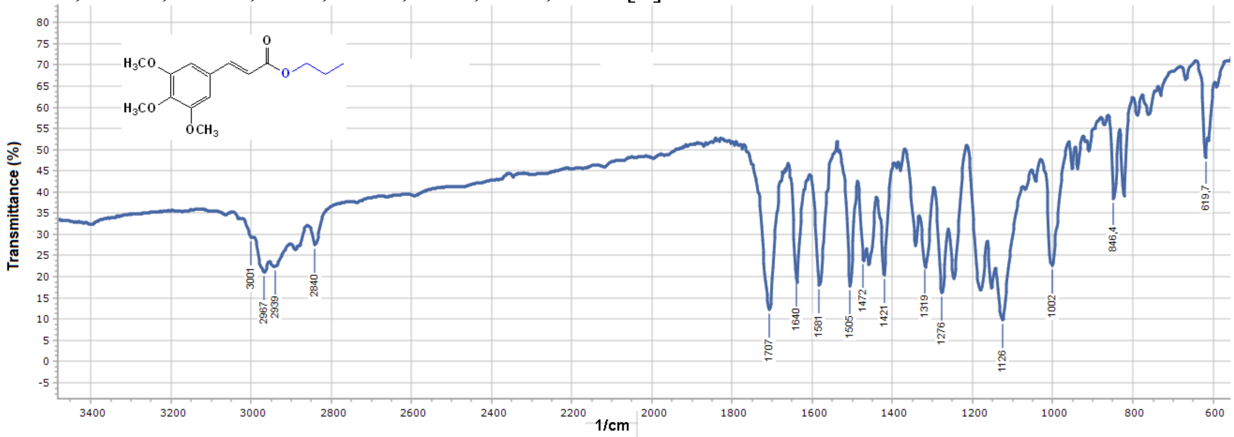

Figure S7: Infrared spectrum (KBr, cm^{-1}) of (*E*)-propyl 3-(3,4,5-trimethoxyphenyl)acrylate (**03**).

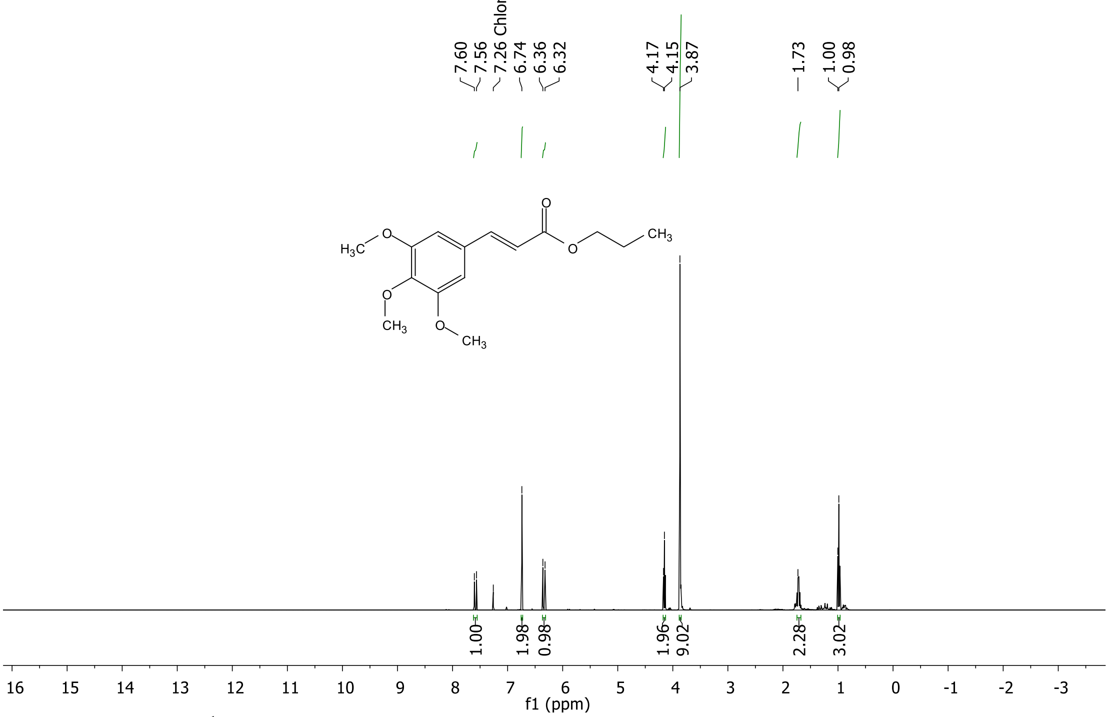

Figure S8: ^{1}H NMR spectrum (400 MHz, $CDCl_3$) of (*E*)-propyl 3-(3,4,5-trimethoxyphenyl)acrylate (**03**).

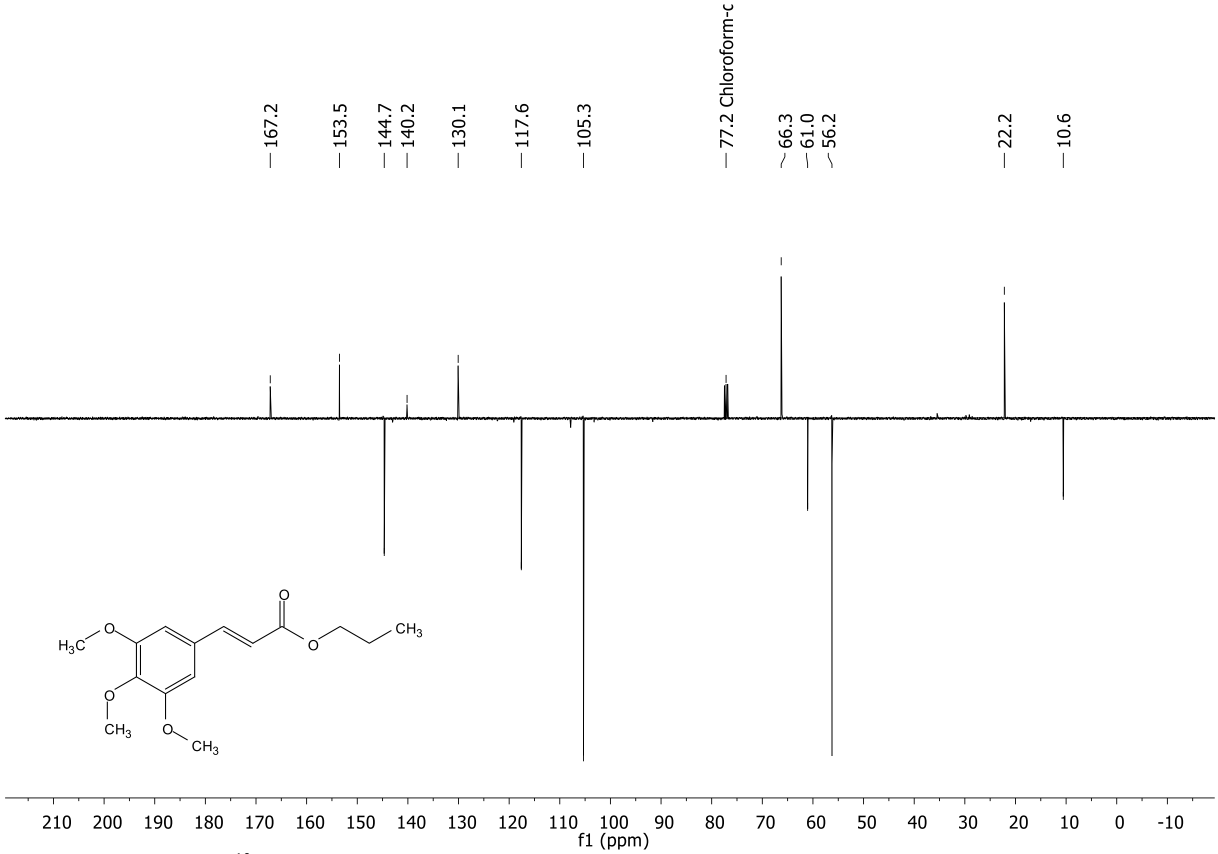

Figure S9: APT-^{13}C NMR spectrum (100 MHz, $CDCl_3$) of (*E*)-propyl 3-(3,4,5-trimethoxyphenyl)acrylate (**03**).

(*E*)-isopropyl 3-(3,4,5-trimethoxyphenyl)acrylate (**4**): brown oil; Yield: 58,8%; Reaction time: 24 h; TLC (7:3 Hex/EtOAc), R_f = 0.60; IR *v*max (KBr, cm^{-1}): 2982, 2941, 2844, 1706, 1636, 1583, 1506, 1467, 1276, 1128, 828; **^{1}H NMR ($CDCl_3$, 400 MHz):** δH 7.57 (*d*; *J*=15.89 Hz; 1H), 6.74 (*s*; 2H), 6.32 (*d*; *J*=15.89 Hz, 1H), 5.16 – 5.10 (*m*, 1H), 3.87 (*s*, 9H), 1.31 (*d*, *J*=6.26 Hz, 6H); **^{13}C NMR ($CDCl_3$, 100 MHz):** δC 166.6, 153.5, 144.4, 140.1, 130.2, 118.2, 105.3, 67.9, 61.1, 56.3, 22.1 [3].

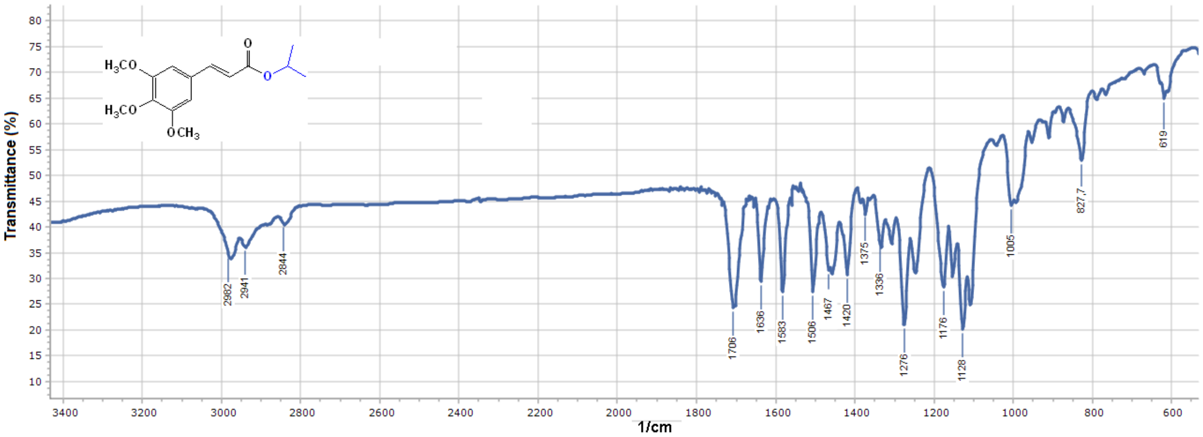

Figure S10: Infrared spectrum (KBr, cm^{-1}) of (*E*)-isopropyl 3-(3,4,5-trimethoxyphenyl)acrylate (**04**).

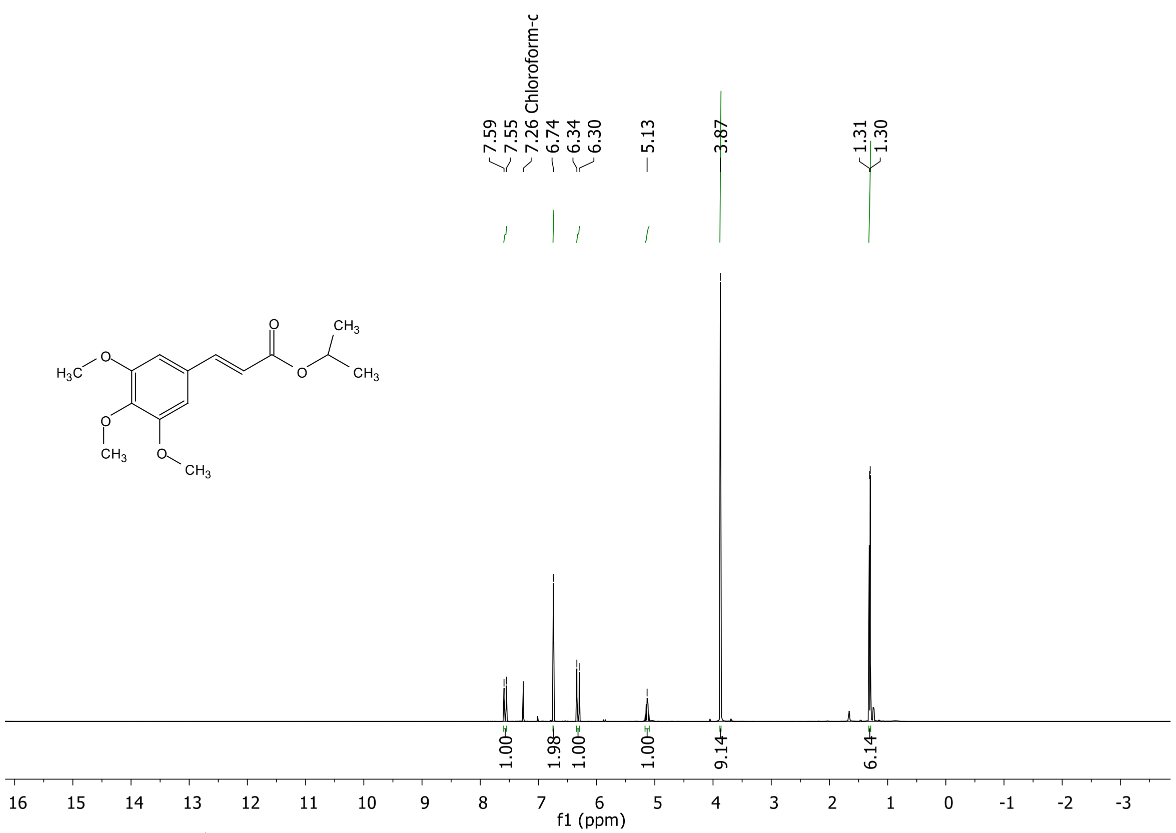

Figure S11: ^{1}H NMR spectrum (400 MHz, $CDCl_3$) of (*E*)-isopropyl 3-(3,4,5-trimethoxyphenyl)acrylate (**04**).

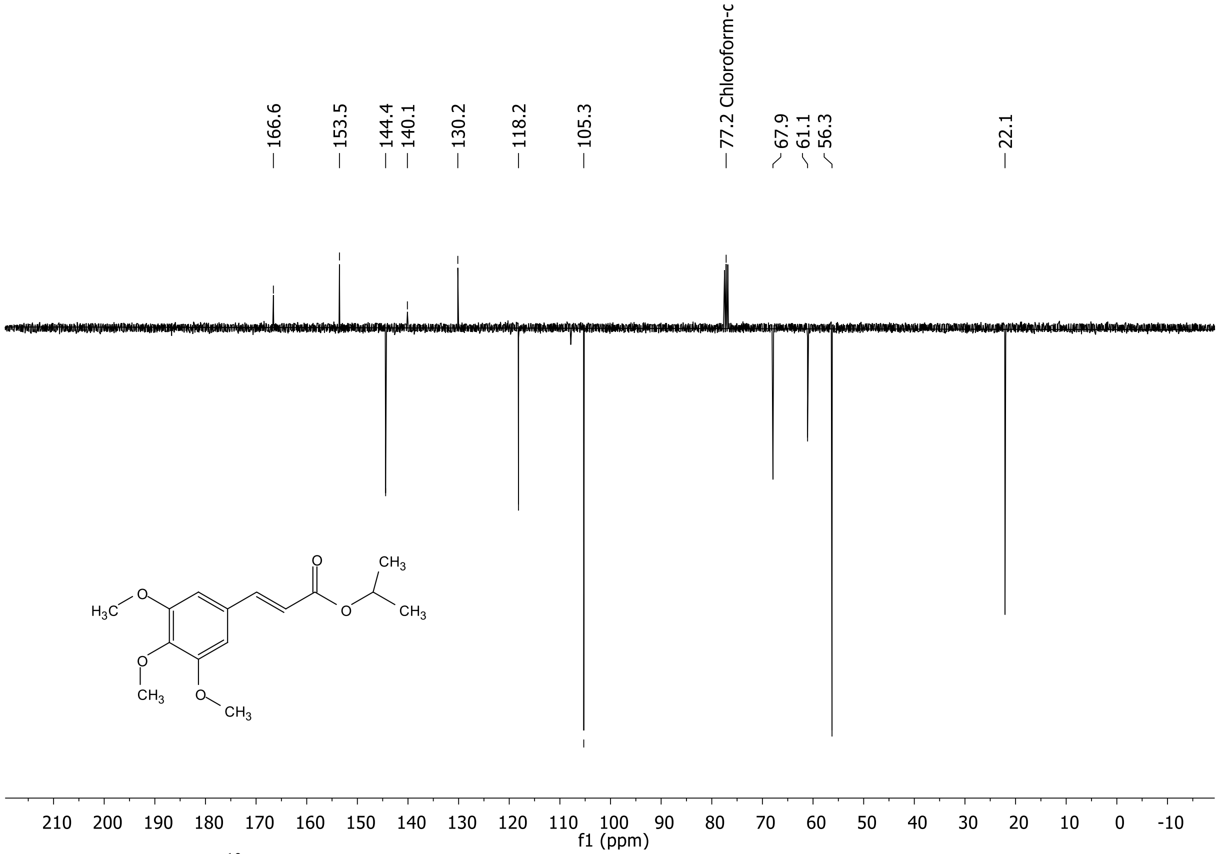

Figure S12: APT-^{13}C NMR spectrum (100 MHz, $CDCl_3$) of (*E*)-isopropyl 3-(3,4,5-trimethoxyphenyl)acrylate (**04**).

(*E*)-pentyl 3-(3,4,5-trimethoxyphenyl)acrylate (**5**): white solid; Yield: 41.9%; Reaction time: 48 h; m.p.: 104-105ºC (lit. 105-106ºC); TLC (7:3, Hex/EtOAc), R_f = 0.65; IR *ν*max (KBr, cm^{-1}): 3006, 2933, 2857, 1707, 1636, 1583, 1508, 1461, 1275, 1127, 820; **^{1}H NMR ($CDCl_3$, 500 MHz):** δH 7.57 (*d*; *J*=15.89 Hz; 1H), 6.74 (*s*; 2H), 6.33 (*d*; *J*=15.89 Hz; 1H), 4.18 (*t*; *J*= 6.70 Hz; 2H), 3.87 (*s*;

9H), 1.71 – 1.66 (*m*; 2H), 1.40 – 1.33 (*m*; 4H), 0.91 (*t*; *J*=6.25 Hz; 3H); **^{13}C NMR ($CDCl_3$, 125 MHz):** δC 167.1, 153.5, 144.6, 140.2, 130.1, 117.6, 105.3, 64.8, 61.0, 56.2, 28.5, 28.2, 22.4, 14.0 [3].

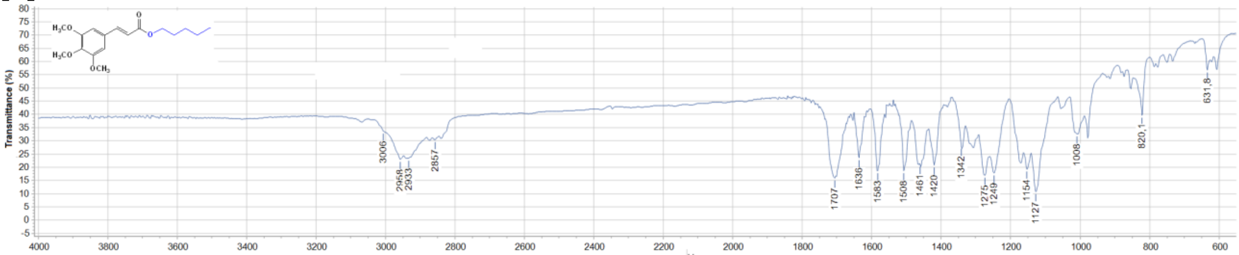

Figure S13: Infrared spectrum (KBr, cm^{-1}) of (*E*)-pentyl 3-(3,4,5-trimethoxyphenyl)acrylate (**05**).

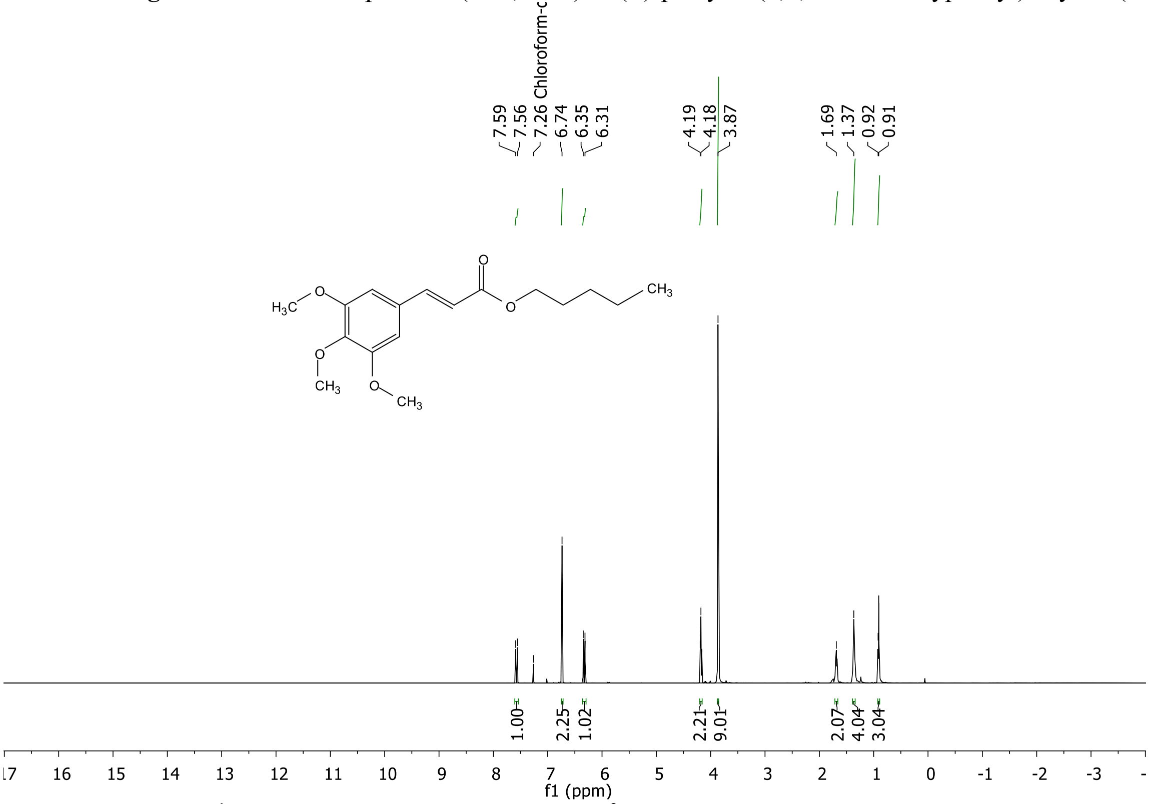

Figure S14: ^{1}H NMR spectrum (500 MHz, $CDCl^3$) of (*E*)-pentyl 3-(3,4,5-trimethoxyphenyl)acrylate (**05**).

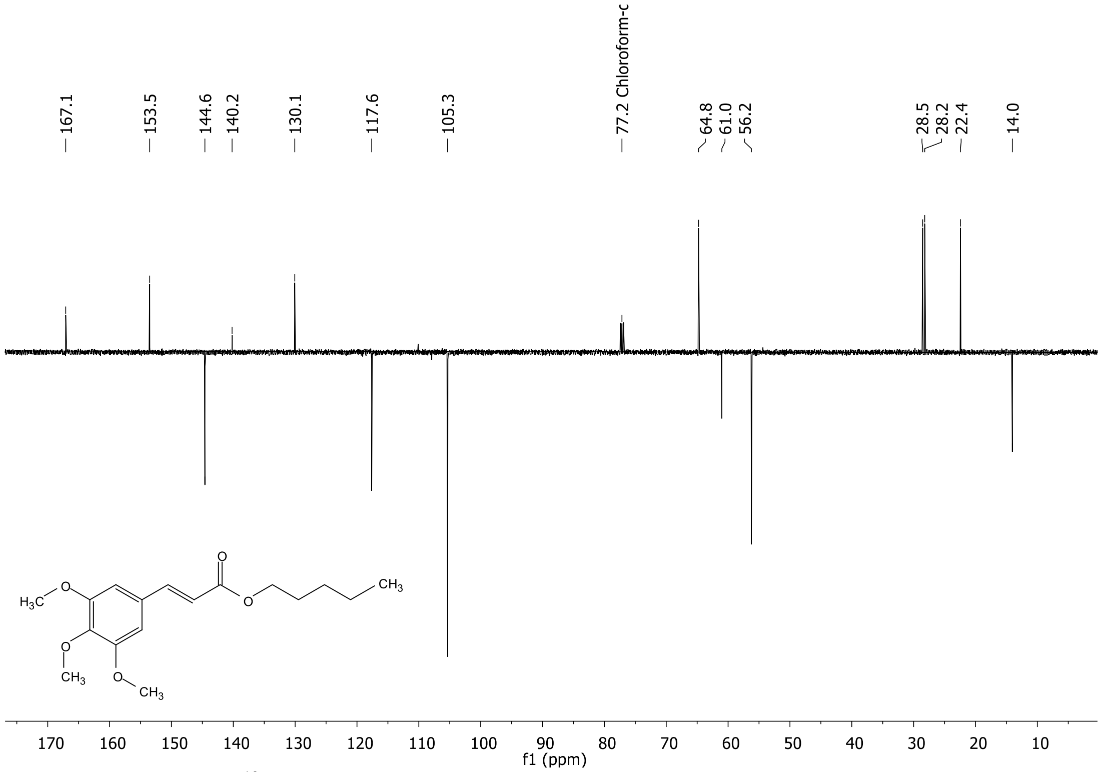

Figure S15: APT-^{13}C NMR spectrum (125 MHz, $CDCl_3$) of (*E*)-pentyl 3-(3,4,5-trimethoxyphenyl)acrylate (**05**).

(*E*)-decyl 3-(3,4,5-trimethoxyphenyl)acrylate (**6**): white solid; Yield: 38.0%; Reaction time: 48 h; m.p.: 35-36ºC (lit. 37-38ºC); TLC (7:3 Hex/EtOAc), R_f = 0.70; IR *v*max (KBr, cm^{-1}): 3001, 2925, 2857, 1714, 1638, 1584, 1508, 1276, 1126, 824; **^{1}H NMR ($CDCl_3$, 400 MHz):** δH 7.58 (*d*; *J*=15.90 Hz; 1H), 6.74 (*s*; 2H), 6.34 (*d*; *J*=15.90 Hz; 1H), 4.20 (*t*; *J*=6.74 Hz; 2H), 3.87 (*s*; 9H), 1.73 – 1.66 (*m*; 2H), 1.40 – 1.26 (*m*; 14H), 0.87 (*t*; *J*= 6.61 Hz; 3H) **^{13}C NMR ($CDCl_3$, 100 MHz):** δC 167.2, 153.6, 144.7, 140.2, 130.1, 117.7, 105.4, 64.9, 61.1, 56.3, 32.1, 29.7, 29.5, 28.9, 26.2, 22.8, 14.3 [3].

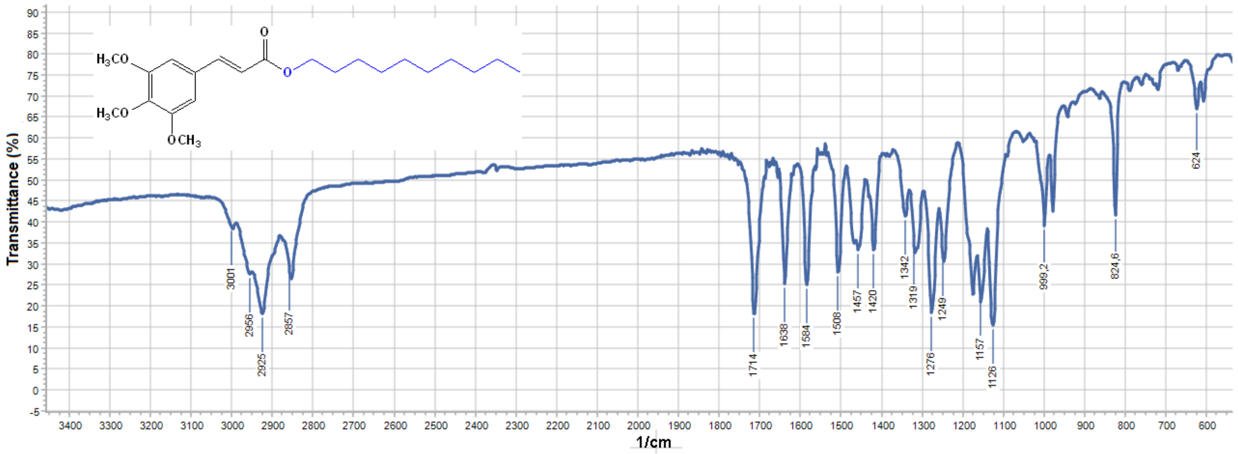

Figure S16: Infrared spectrum (KBr, cm^{-1}) of (*E*)-decyl 3-(3,4,5-trimethoxyphenyl)acrylate (**06**).

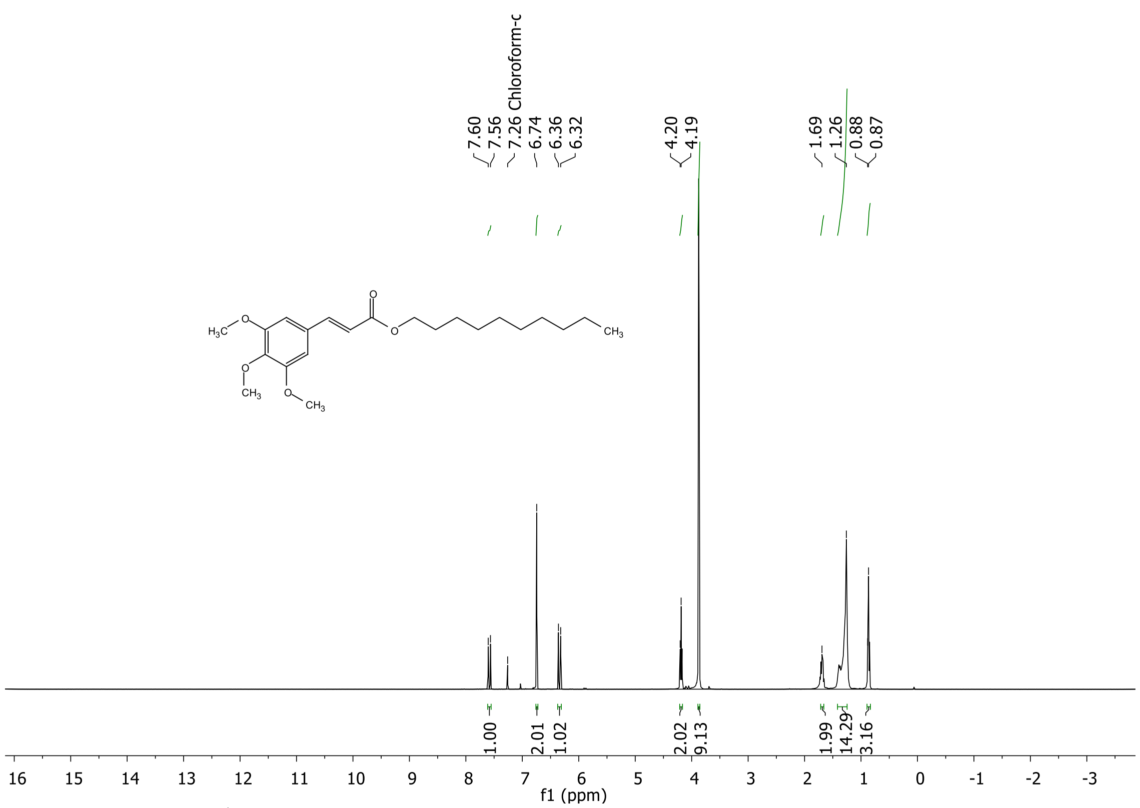

Figure S17: ^{1}H NMR spectrum (400 MHz, $CDCl_3$) of (*E*)-decyl 3-(3,4,5-trimethoxyphenyl)acrylate (**06**).

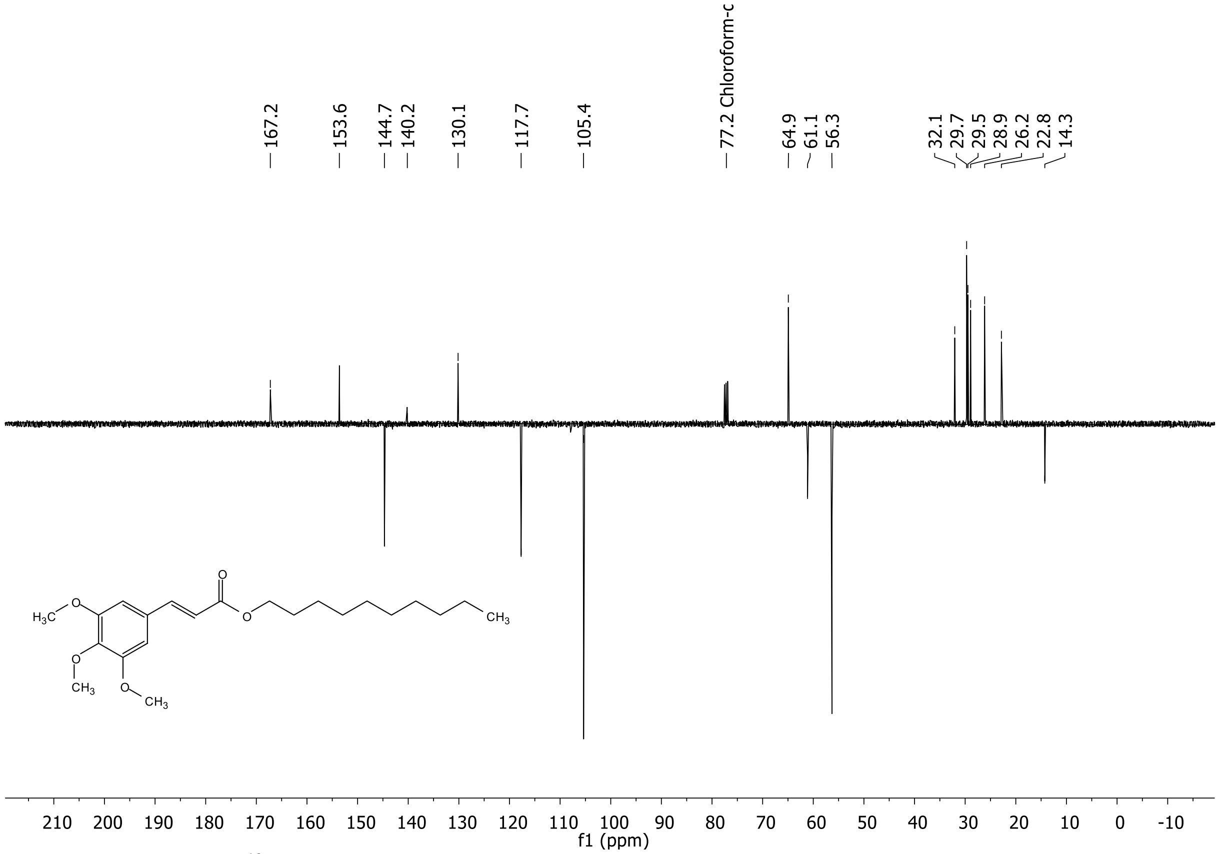

Figure S18: APT-^{13}C NMR spectrum (100 MHz, $CDCl_3$) of (*E*)-decyl 3-(3,4,5-trimethoxyphenyl)acrylate (**06**).

(*E*)-4-methoxybenzyl 3-(3,4,5-trimethoxyphenyl)acrylate (**7**): white solid; Yield: 47.5%; Reaction time: 48 h; m.p.: 70-71ºC (lit. 65-67ºC); TLC (7:3 Hex/EtOAc), R_f = 0.58; IR *v*max (KBr, cm^{-1}): 3036, 2942, 2838, 1718, 1640, 1582, 1515, 1467, 1278, 1133, 817; **^{1}H NMR ($CDCl_3$, 500 MHz):** δH 7.61 (*d*; *J*=15.92 Hz; 1H), 7.35 (*d*; *J*= 8.67; 2H), 6.91 (*d*; *J*= 8.67; 2H), 6.73 (*s*; 2H), 6.37 (*d; J*=15.92 Hz; 1H), 5.18 (*s*; 2H), 3.87 (*s*; 9H), 3.81 (*s;* 3H); **^{13}C NMR ($CDCl_3$, 125 MHz):** δC

166.9, 159.8, 153.6, 145.1, 140.3, 130.3, 130.0, 128.3, 117.4, 114.1, 105.4, 66.4, 61.1, 56.3, 55.5 [4].

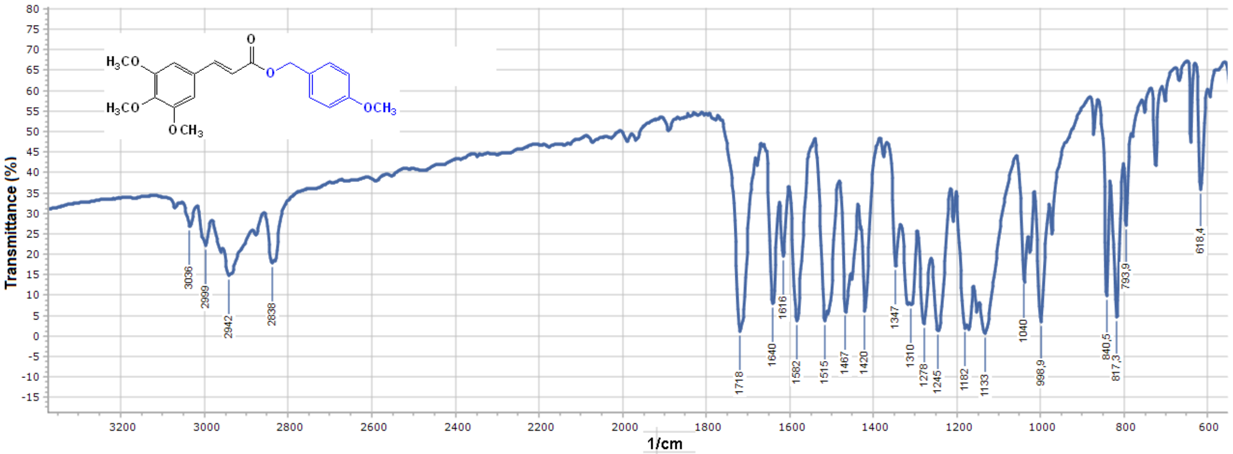

Figure S19: Infrared spectrum (KBr, cm^{-1}) of (*E*)-4-methoxybenzyl 3-(3,4,5-trimethoxyphenyl)acrylate (**07**).

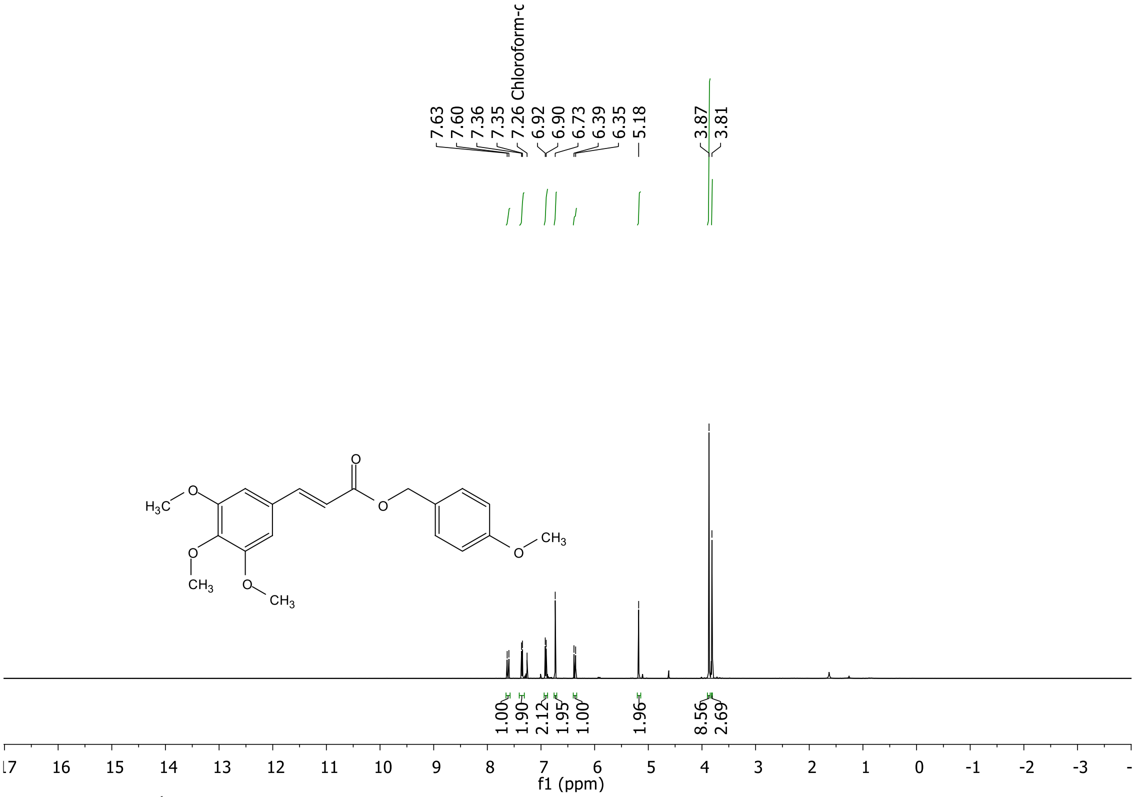

Figure S20: ^{1}H NMR spectrum (500 MHz, $CDCl_3$) of (*E*)-4-methoxybenzyl 3-(3,4,5-trimethoxyphenyl)acrylate (**07**).

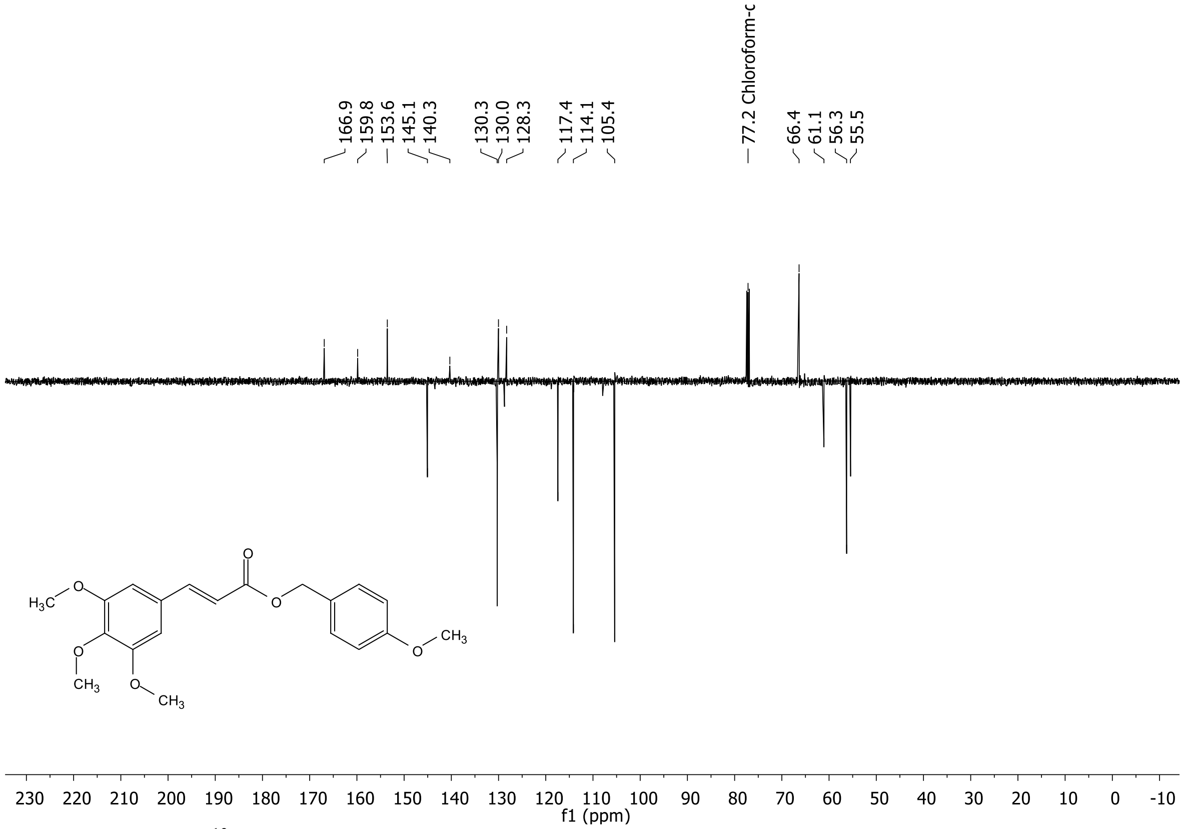

Figure S21: APT-^{13}C NMR spectrum (125 MHz, $CDCl_3$) of (*E*)-4-methoxybenzyl 3-(3,4,5-trimethoxyphenyl)acrylate (**07**).

(*E*)-3-methoxybenzyl 3-(3,4,5-trimethoxyphenyl)acrylate (**8**): colorless oil; Yield: 26.7%; Reaction time: 48 h; TLC (7:3 Hex/EtOAc), $R_f = 0.58$; IR *v*max (KBr, cm^{-1}): 3002, 2939, 2840, 1713, 1636, 1584, 1505, 1459, 1278, 1136, 846; **^{1}H NMR ($CDCl_3$, 400 MHz):** δH 7.64 (*d*; *J*=15.90 Hz; 1H), 7.31 (*t*; *J*= 7.9 Hz; 1H), 7.02 – 6.95 (*m*; 2H), 6.90 – 6.86 (*m;* 1H), 6.75 (*s*; 2H), 6.40 (*d; J*=15.90 Hz; 1H), 5.22 (*s*; 2H), 3.88 (*s*; 9H), 3.83 (*s;* 3H); **^{13}C NMR ($CDCl_3$, 100 MHz):** δC 166.8, 159.9, 153.6, 145.3, 140.3, 137.7, 130.0, 129.8, 120.6, 117.2, 114.0, 113.8, 105.4, 66.4, 61.1, 56.2, 55.4. HRMS (MALDI TOF/TOF) calculated for $C_{20}H_{22}O_6$ *m/z* $[M]^+$: 358.1416, found 358,1416.

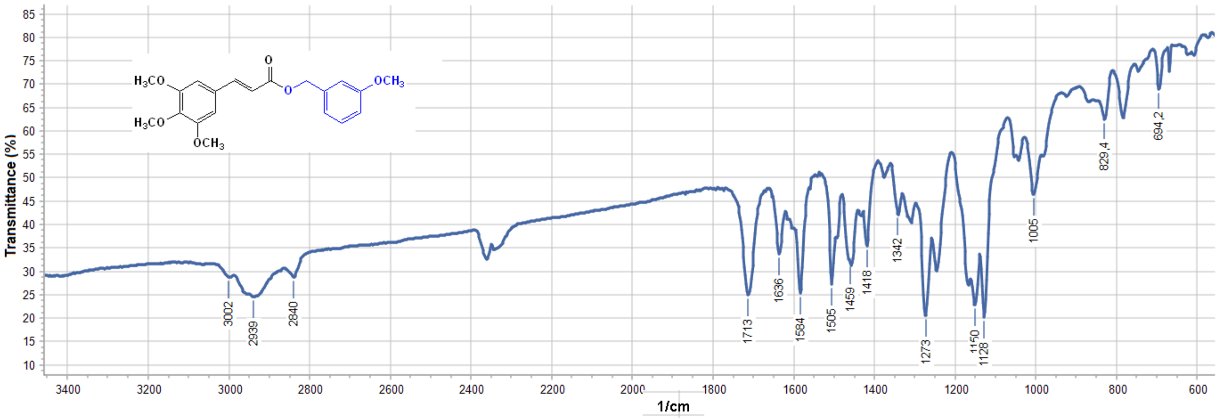

Figure S22: Infrared spectrum (KBr, cm^{-1}) of (*E*)-3-methoxybenzyl 3-(3,4,5-trimethoxyphenyl)acrylate (**08**).

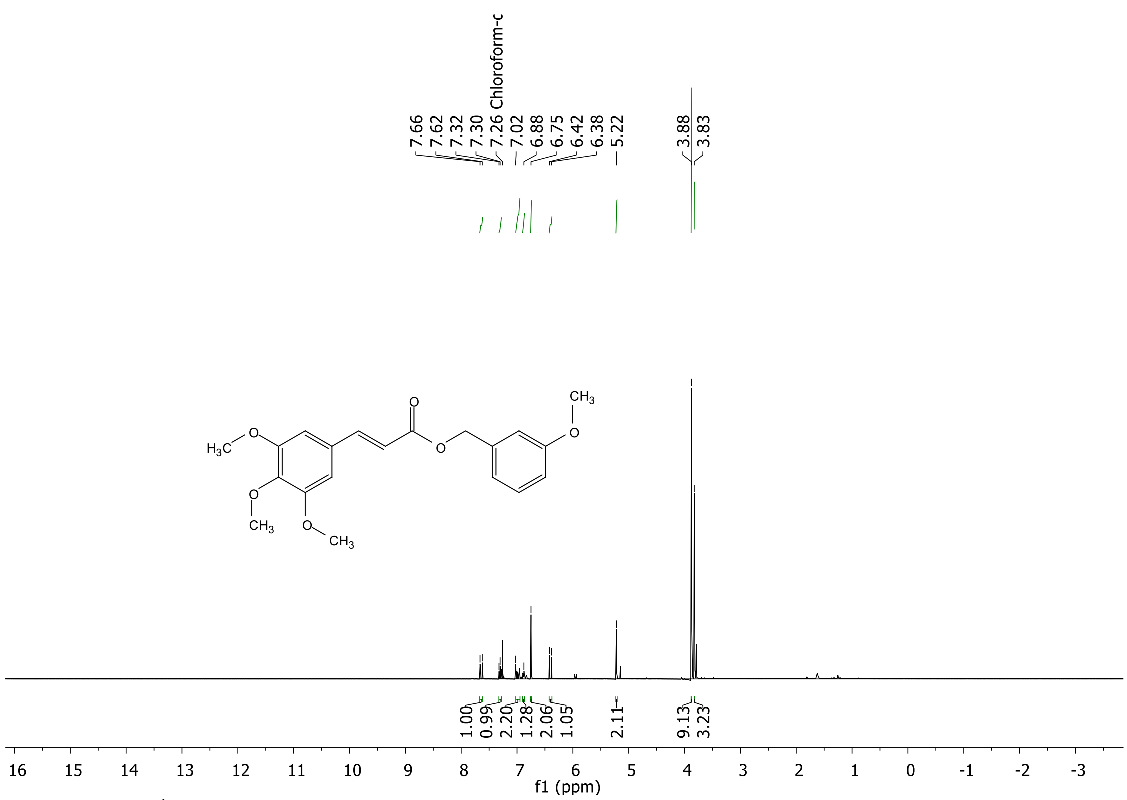

Figure S23: ^{1}H NMR spectrum (400 MHz, $CDCl_3$) of (*E*)-3-methoxybenzyl 3-(3,4,5-trimethoxyphenyl)acrylate (**08**).

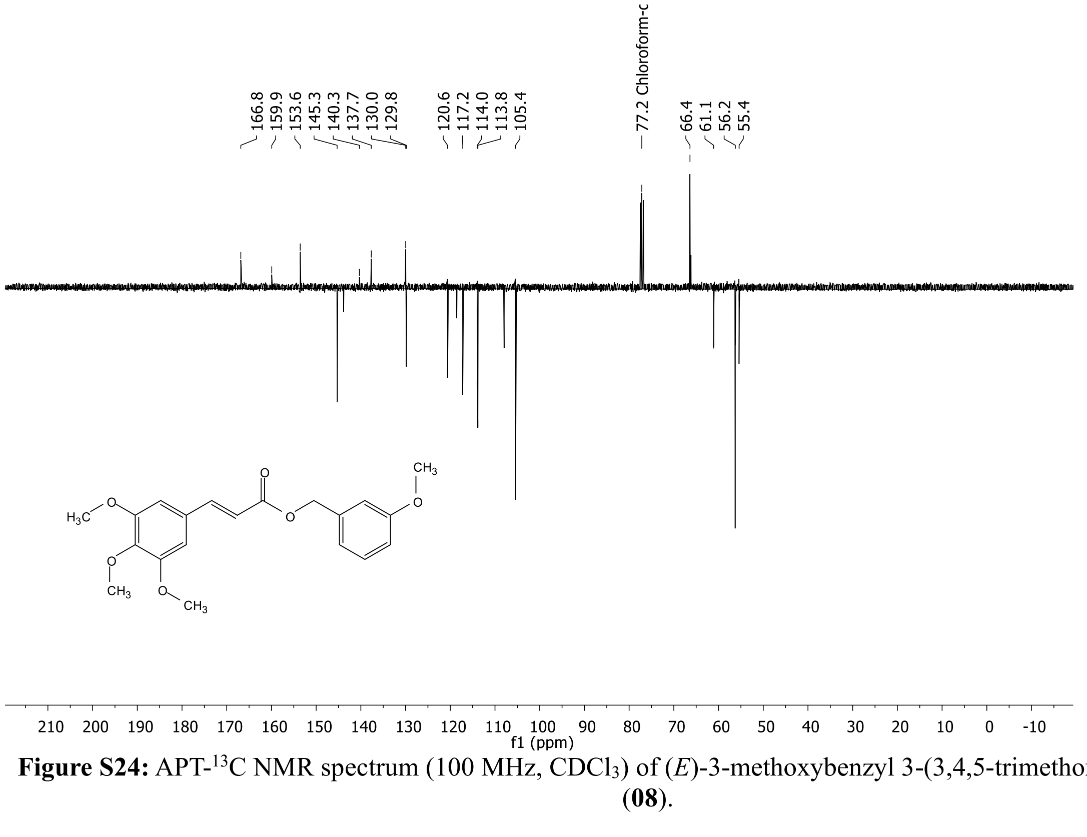

Figure S24: APT-^{13}C NMR spectrum (100 MHz, $CDCl_3$) of (*E*)-3-methoxybenzyl 3-(3,4,5-trimethoxyphenyl)acrylate (**08**).

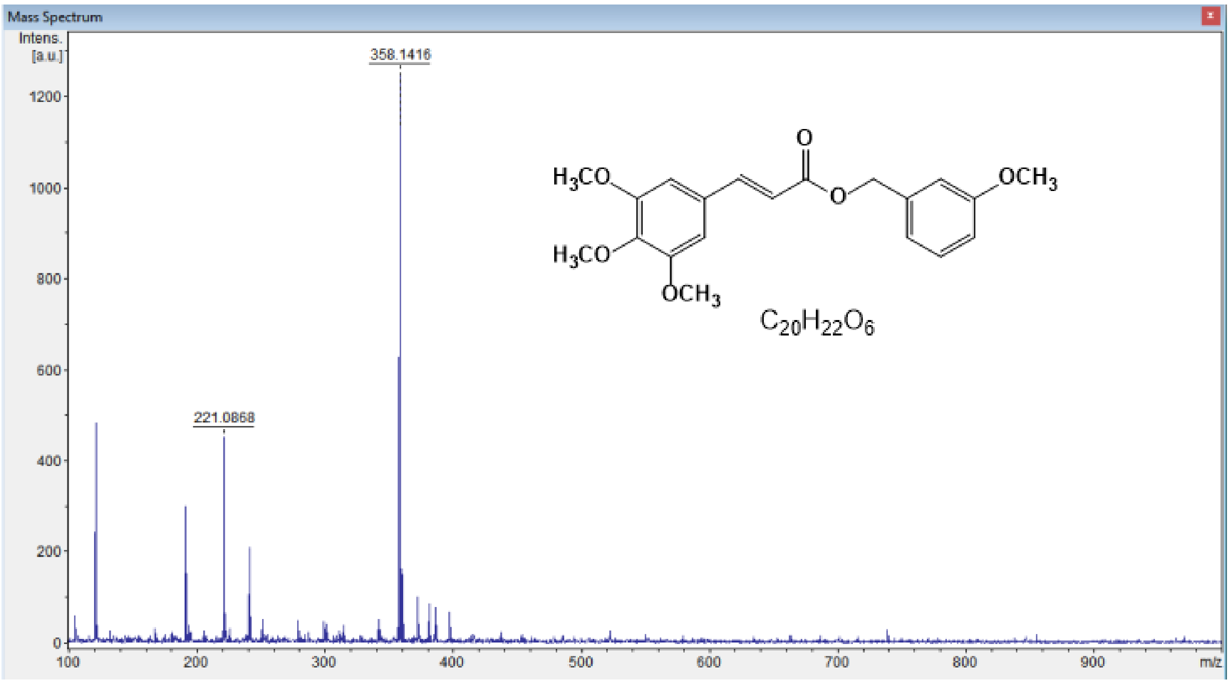

Figure S25: HRMS spectrum (MALDI TOF/TOF) of (*E*)-3-methoxybenzyl 3-(3,4,5-trimethoxyphenyl)acrylate (**08**).

(*E*)-4-methylbenzyl 3-(3,4,5-trimethoxyphenyl)acrylate (**9**): colorless oil; Yield: 62.8%; Reaction time: 24 h; TLC (7:3 Hex/EtOAc), $R_f = 0.62$; IR *v*max (KBr, cm^{-1}): 3010, 2932, 2837, 1716, 1640, 1582, 1509, 1465, 1278, 1136, 846; **^{1}H NMR ($CDCl_3$, 500 MHz):** δH 7.63 (*d*; *J*=15.92 Hz; 1H), 7.31 (*d*; *J*=7.98 Hz; 2H), 7.19 (*d*; *J*= 7.82 Hz; 2H) 6.74 (*s*; 2H), 6.38 (*d; J*=15.92 Hz; 1H), 5.21 (*s*; 2H), 3.87 (*s*; 9H), 2.36 (*s;* 3H); **^{13}C NMR ($CDCl_3$, 125 MHz):** δC 166.9, 153.3, 145.1, 140.3, 138.3, 133.1, 130.0, 129.4, 128.6, 117.4, 105.4, 66.5, 61.1, 56.3, 21.3. HRMS (MALDI TOF/TOF) calculated for $C_{20}H_{22}O_5$ *m/z* $[M]^+$: 342.1467, found 342.1475.

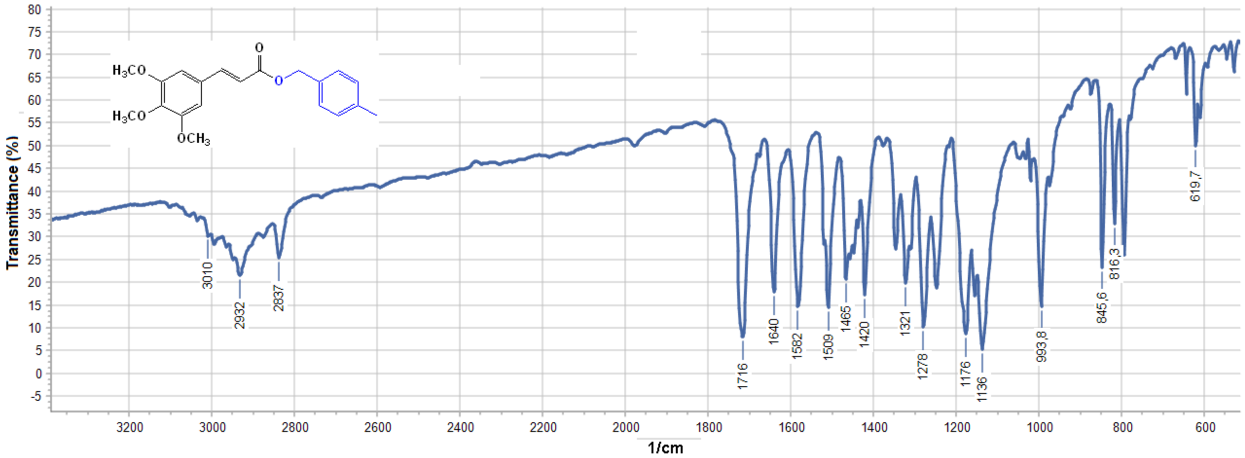

Figure S26: Infrared spectrum (KBr, cm^{-1}) of (*E*)-4-methylbenzyl 3-(3,4,5-trimethoxyphenyl)acrylate (**09**).

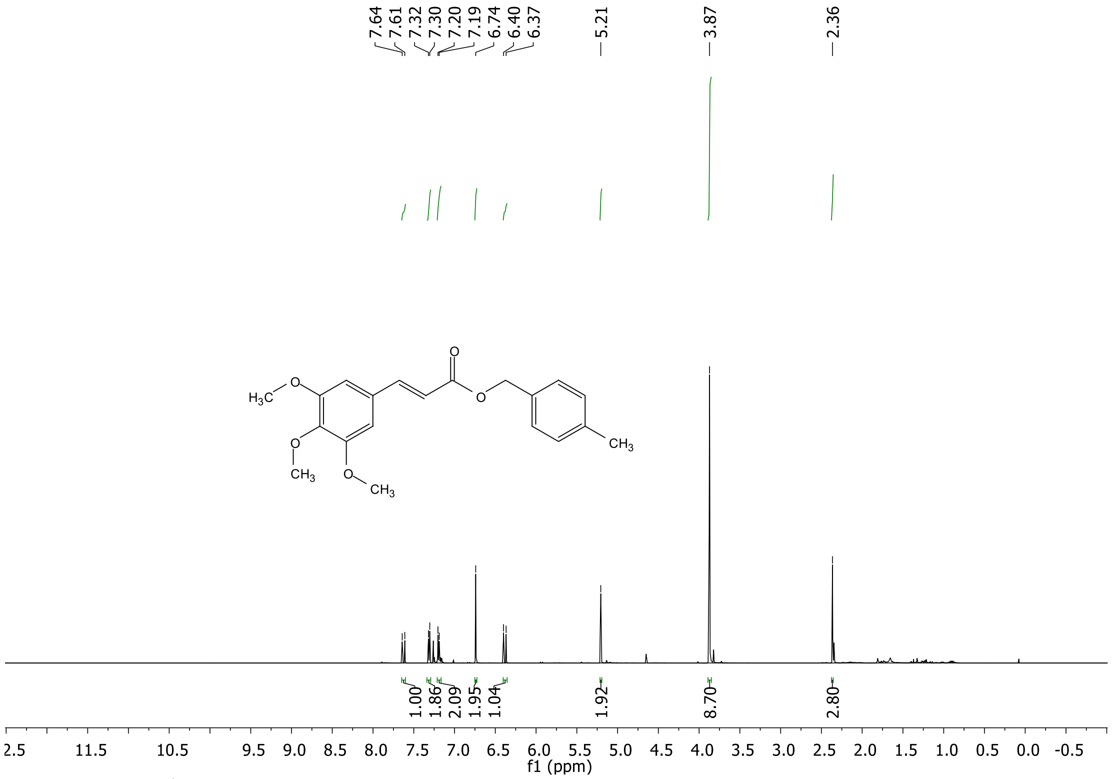

Figure S27: ^{1}H NMR spectrum (500 MHz, $CDCl_3$) of (*E*)-4-methylbenzyl 3-(3,4,5-trimethoxyphenyl)acrylate (**09**).

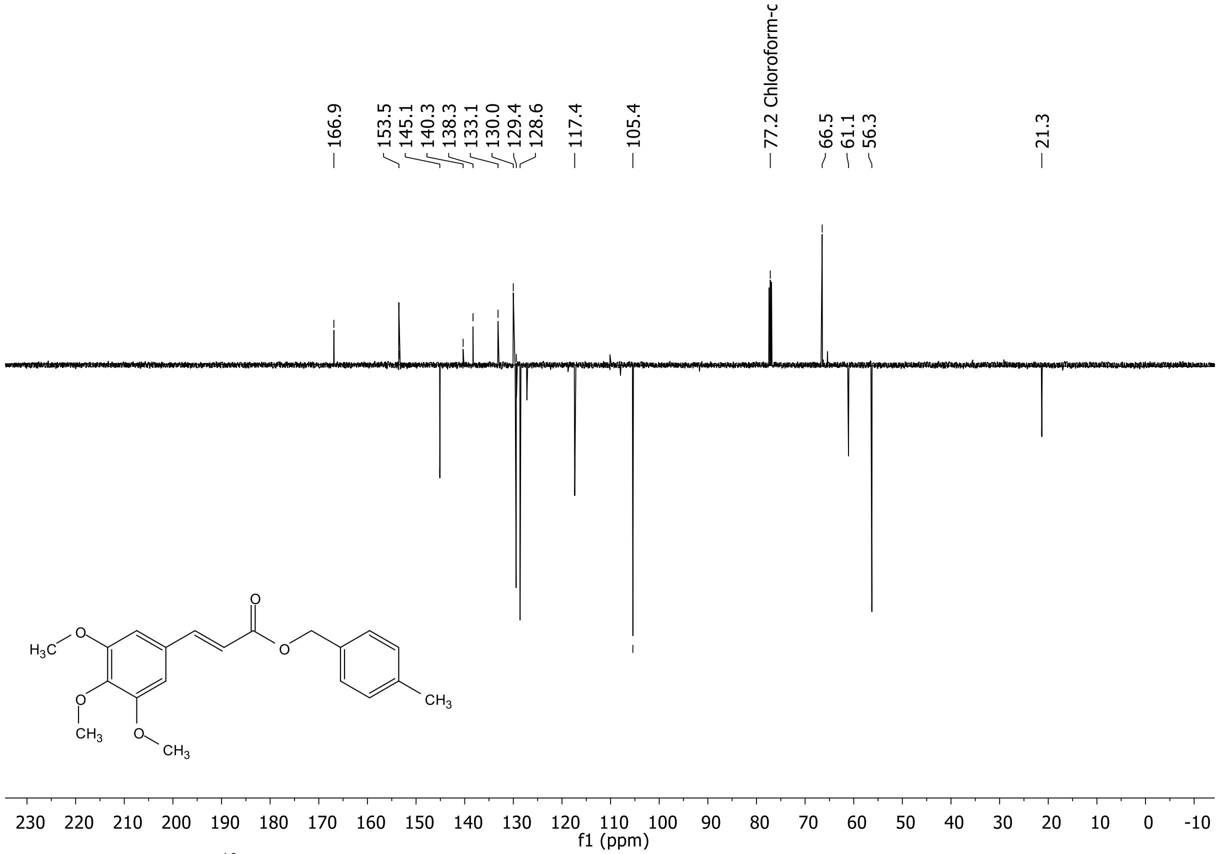

Figure S28: APT-^{13}C NMR spectrum (125 MHz, $CDCl_3$) of (*E*)-4-methylbenzyl 3-(3,4,5-trimethoxyphenyl)acrylate (**09**).

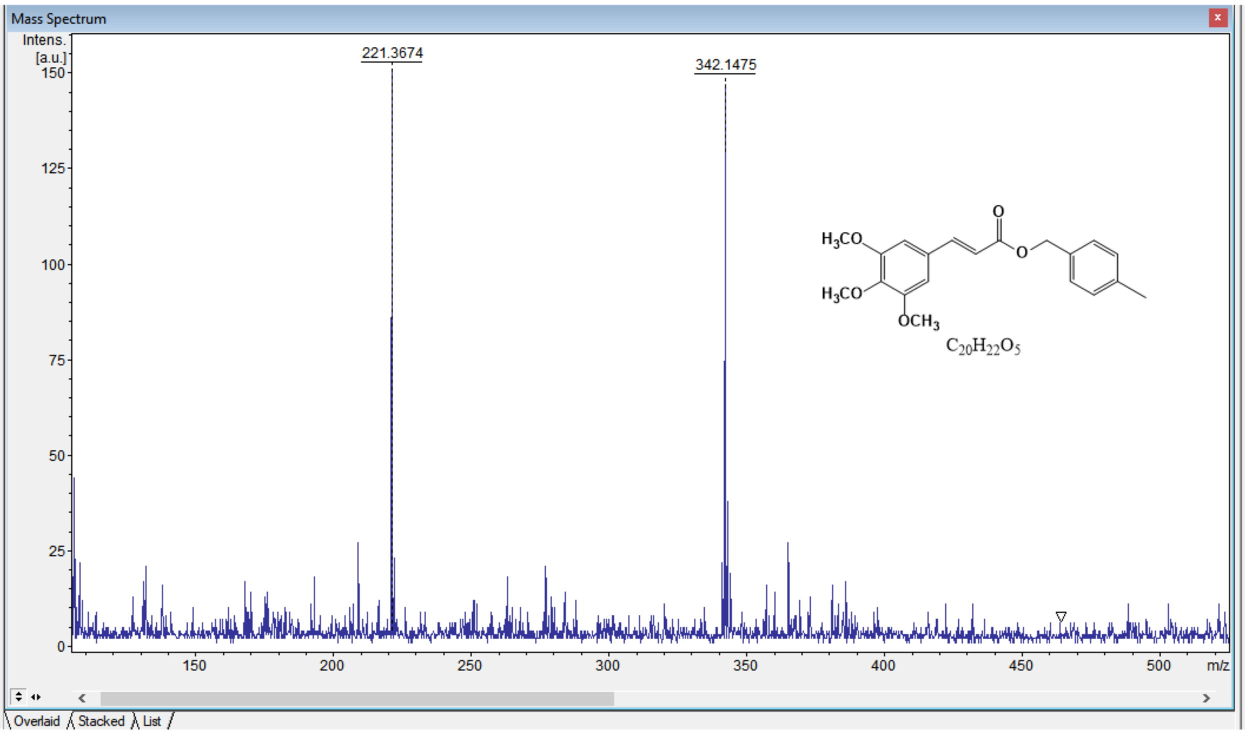

Figure S29: HRMS spectrum (MALDI TOF/TOF) of (*E*)-4-methylbenzyl 3-(3,4,5-trimethoxyphenyl)acrylate (**09**).

(*E*)-benzyl 3-(3,4,5-trimethoxyphenyl)acrylate (**10**): white solid; Yield: 48.6%; Reaction time: 24 h; m.p.: 82-83ºC (lit. 85-86ºC); TLC (7:3 Hex/EtOAc), R_f = 0.55; IR *v*max (KBr, cm^{-1}): 3021, 2935, 2842, 1704, 1638, 1582, 1506, 1472, 1276, 1127, 827; **^{1}H NMR ($CDCl_3$, 400 MHz):** δH 7.64 (*d*; *J*=15.90 Hz; 1H), 7.43 – 7.37 (*m*; 5H), 6.75 (*s*; 2H), 6.40 (*d; J*=15;90 Hz; 1H), 5.25 (*s*; 2H), 3.88 (*s*; 9H); **^{13}C NMR ($CDCl_3$, 100 MHz):** δC 166.8, 153.5, 145.2, 140.2, 136.1, 129.9, 128.6, 128.3, 127.0, 117.1, 105.3, 66.4, 61.0, 56.2 [5]

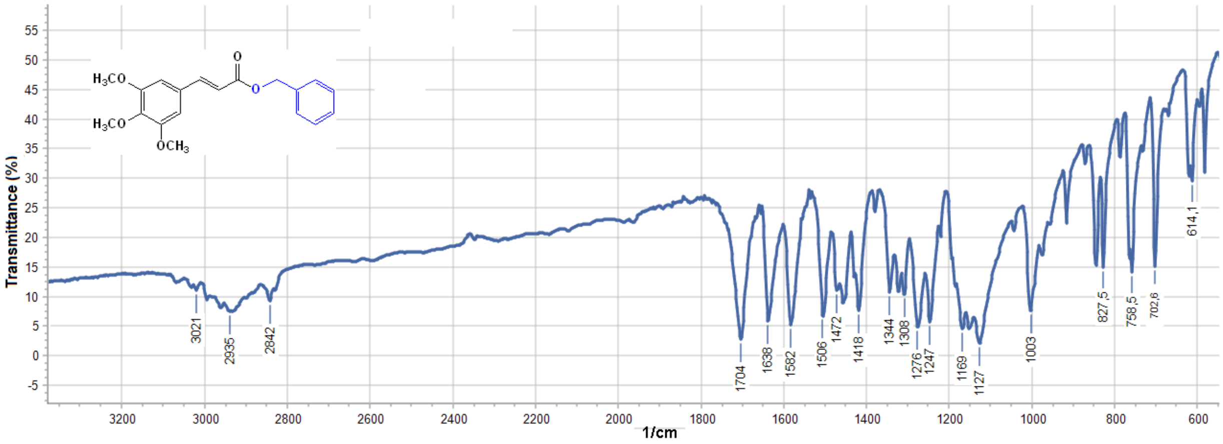

Figure S30: Infrared spectrum (KBr, cm^{-1}) of (*E*)-benzyl 3-(3,4,5-trimethoxyphenyl)acrylate (**10**).

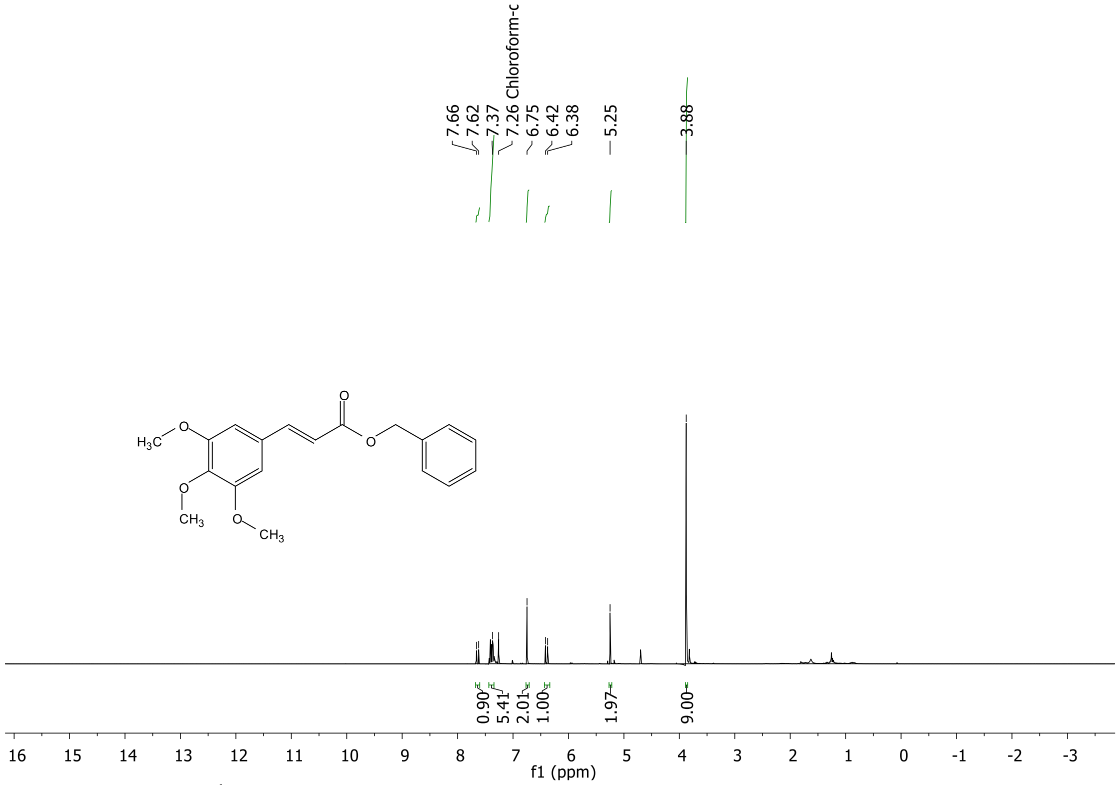

Figure S31: ^{1}H NMR spectrum (400 MHz, $CDCl_3$) of (*E*)-benzyl 3-(3,4,5-trimethoxyphenyl)acrylate (**10**).

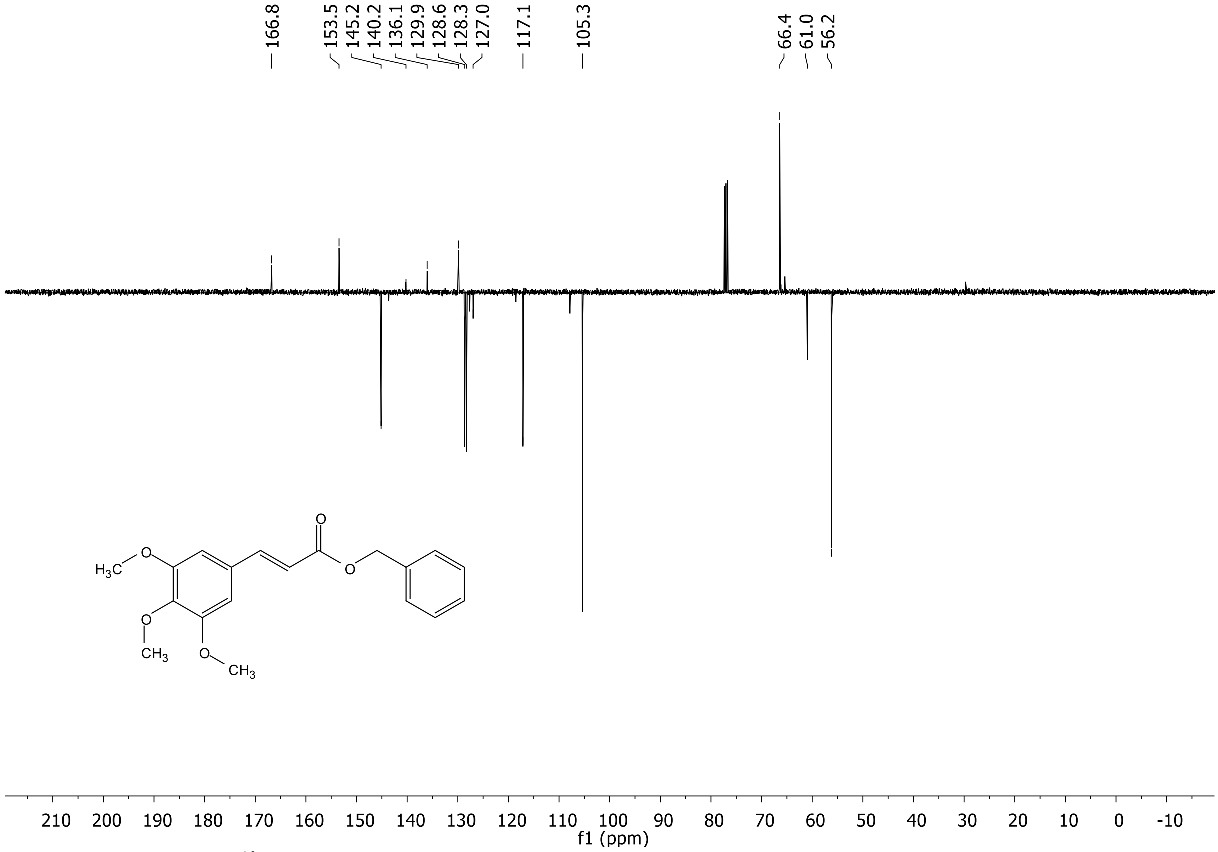

Figure S32: APT-^{13}C NMR spectrum (100 MHz, $CDCl_3$) of (*E*)-benzyl 3-(3,4,5-trimethoxyphenyl)acrylate (**10**).

(*E*)-furan-2-ylmethyl 3-(3,4,5-trimethoxyphenyl)acrylate (**11**): brown oil; Yield: 41.2%; Reaction time: 48 h; TLC (7:3 Hex/EtOAc), $R_f = 0.60$; IR *v*max (KBr, cm^{-1}): 3002, 2945, 2842, 1713, 1638, 1583, 1505, 1459, 1275, 1127, 827; **^{1}H NMR ($CDCl_3$, 500 MHz):** δH 7.62 (*d*; *J*=15.91 Hz; 1H), 7.45 – 7.44 (*m*; 1H) 6.74 (*s*; 2H), 6.46 – 6.45 (*m*; 1H), 6.38 – 6.35 (*m;* 2H), 5.19 (*s*; 2H), 3.86 (*s*; 9H); **^{13}C NMR ($CDCl_3$, 125 MHz)**: δC 166.6, 153.6, 149.7, 145.5, 143.5, 140.4, 129.9, 116.9, 110.8, 110.7, 105.5, 61.1, 58.3, 56.3 [6].

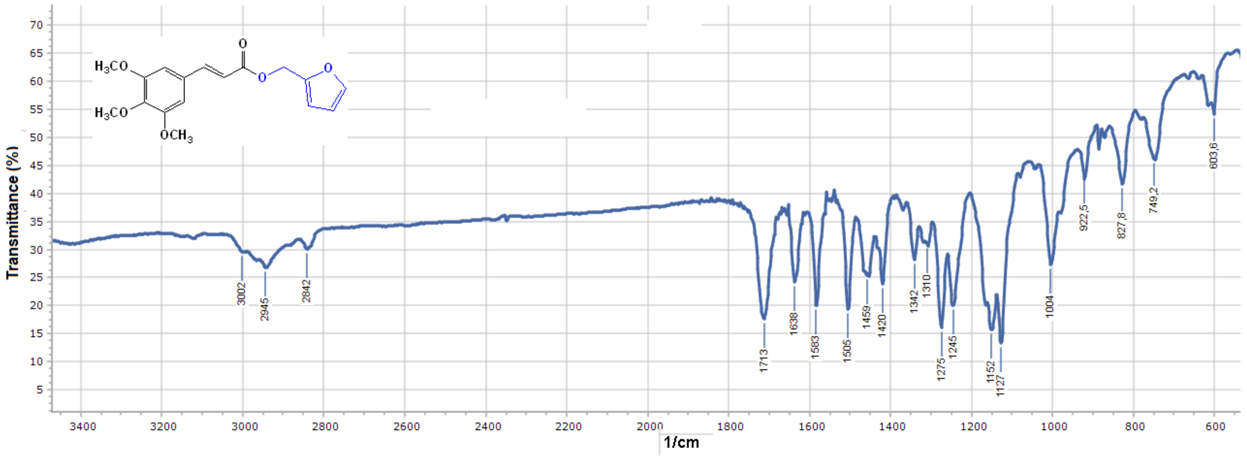

Figure S33: Infrared spectrum (KBr, cm^{-1}) of (*E*)-furan-2-ylmethyl 3-(3,4,5-trimethoxyphenyl)acrylate (**11**).

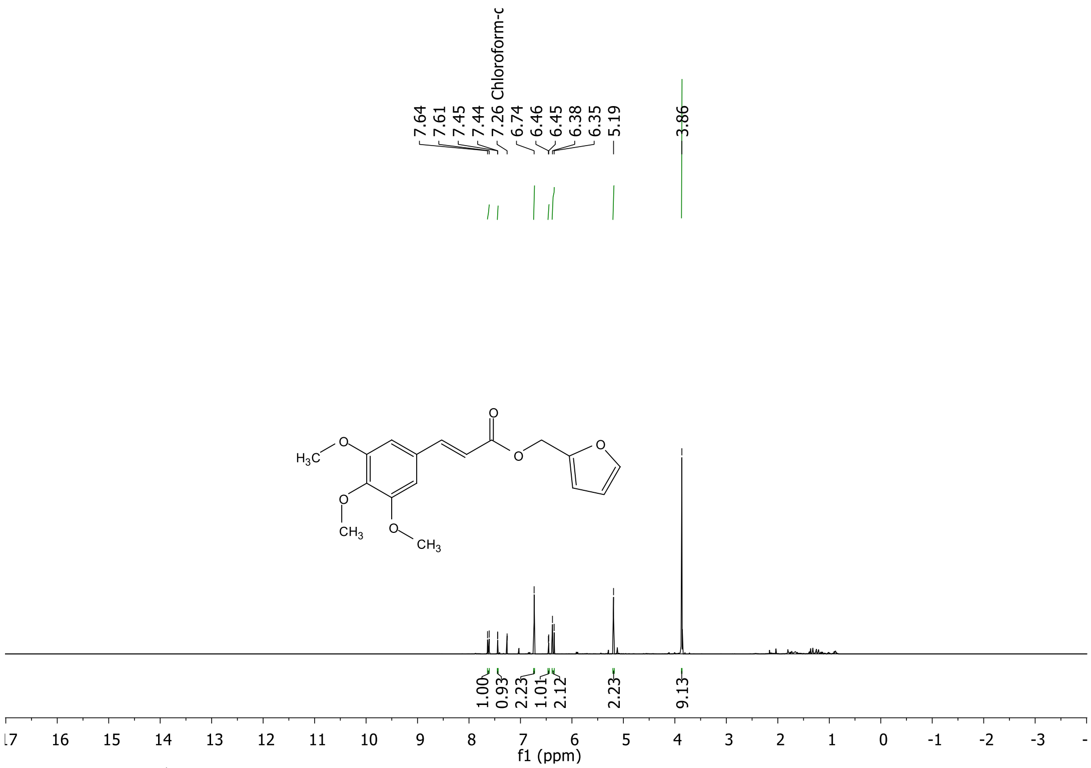

Figure S34: ^{1}H NMR spectrum (500 MHz, $CDCl_3$) of (*E*)-furan-2-ylmethyl 3-(3,4,5-trimethoxyphenyl)acrylate (**11**).

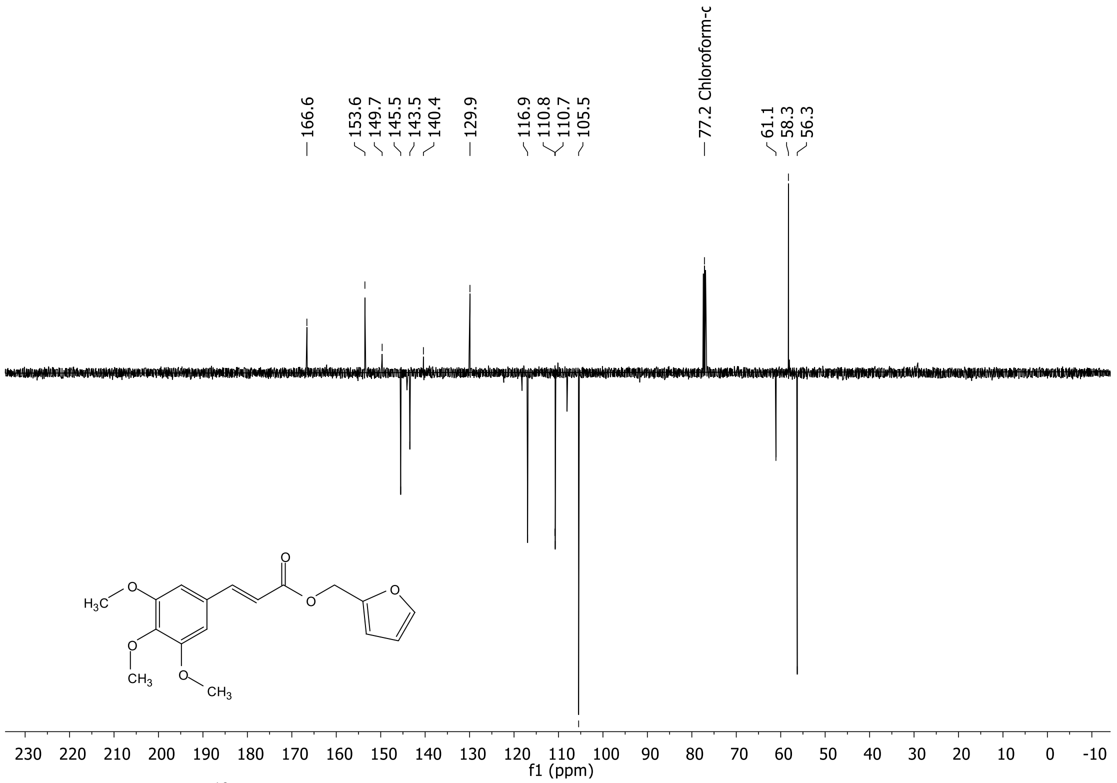

Figure S35: APT-^{13}C NMR spectrum (125 MHz, $CDCl_3$) of (*E*)-furan-2-ylmethyl 3-(3,4,5-trimethoxyphenyl)acrylate (**11**).

(*S*,*E*)-(4-(prop-1-en-2-yl)cyclohex-1-en-1-yl)methyl 3-(3,4,5-trimethoxyphenyl)acrylate (**12**): colorless oil; Yield: 47.6%; Reaction time: 24 h; TLC (7:3 Hex/EtOAc), R_f = 0.70; IR *v*max (KBr, cm^{-1}): 3006, 2967, 2838, 1709, 1638, 1582, 1508, 1461, 1274, 1128, 826; **^{1}H NMR ($CDCl_3$, 400 MHz):** δH 7.60 (*d*; *J*=15.90 Hz; 1H), 6.75 (*s*; 2H), 6.36 (*d; J*= 15.90 Hz; 1H), 5.80 (*sl*; 1H), 4.72 (*s;* 2H), 4.60 (*s*; 2H), 3.88 (*s*; 9H), 2.15 (*m*; 4H), 2.02 (*m;* 1H), 1.86 (*m*; 1H), 1.74 (*s*; 3H), 1.52 (*m;* 1H); **^{13}C NMR ($CDCl_3$, 100 MHz):** δC 167.0, 153.6, 149.7, 144.9, 140.3, 132.8, 130.1, 126.0, 117.5, 108.9, 105.4, 68.7, 61.1, 56.3, 41.0, 30.6, 27.5, 26.6, 20.8. HRMS (MALDI TOF/TOF) calculated for $C_{22}H_{28}O_5$ *m/z* $[M]^+$: 372.1937, found 372.1957.

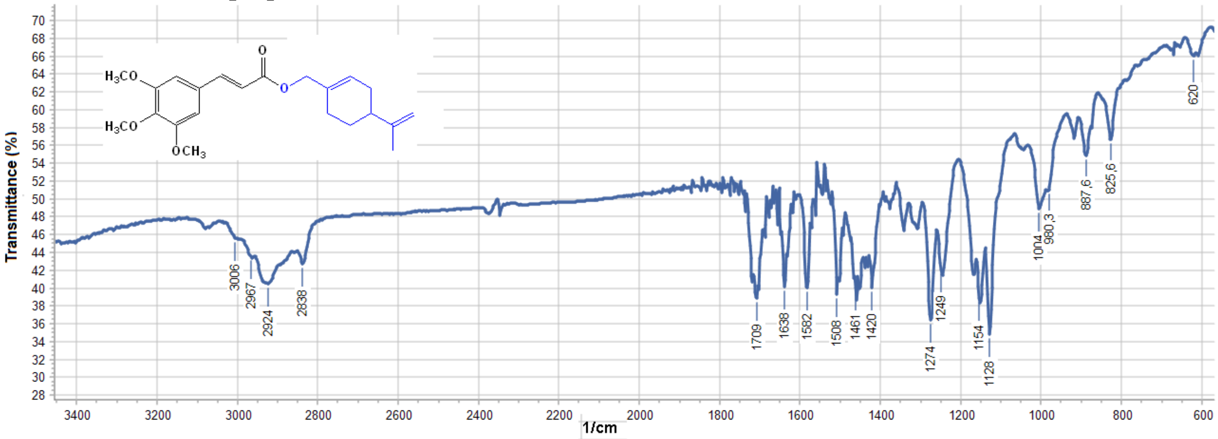

Figure S36: Infrared spectrum (KBr, cm^{-1}) of (*S*, *E*)-(4-(prop-1-en-2-yl)cyclohex-1-en-1-yl)methyl 3-(3,4,5-trimethoxyphenyl)acrylate (**12**).

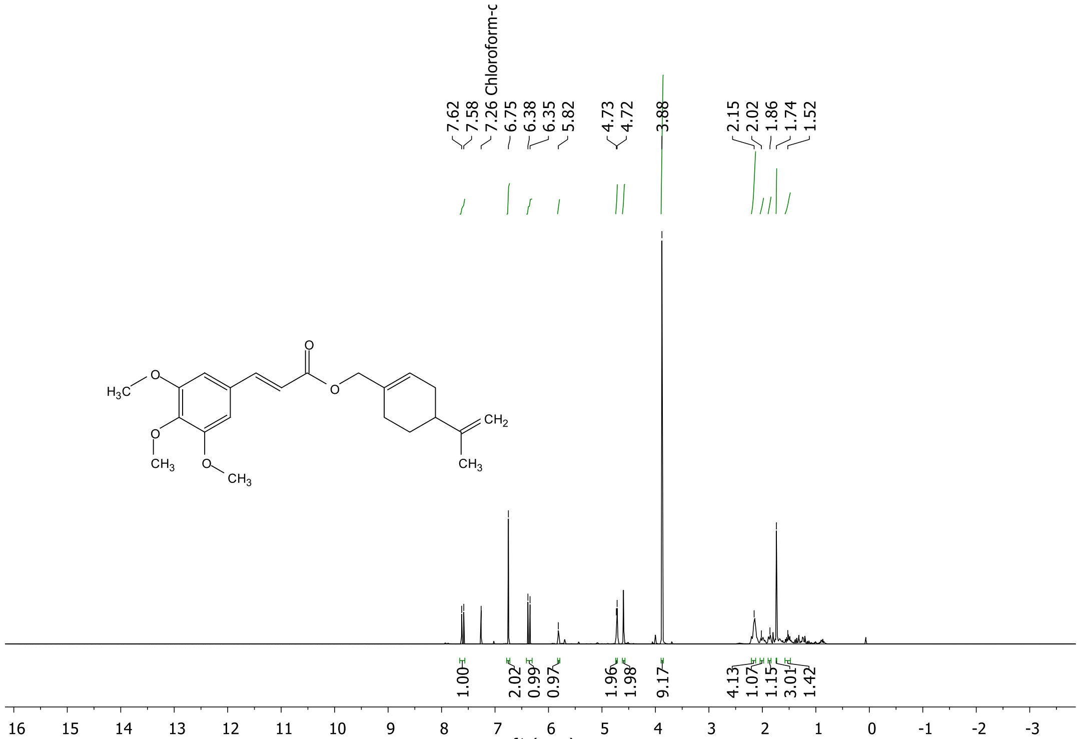

Figure S37: ^{1}H NMR spectrum (400 MHz, $CDCl_3$) of (*S*,*E*)-(4-(prop-1-en-2-yl)cyclohex-1-en-1-yl)methyl 3-(3,4,5-trimethoxyphenyl)acrylate (**12**).

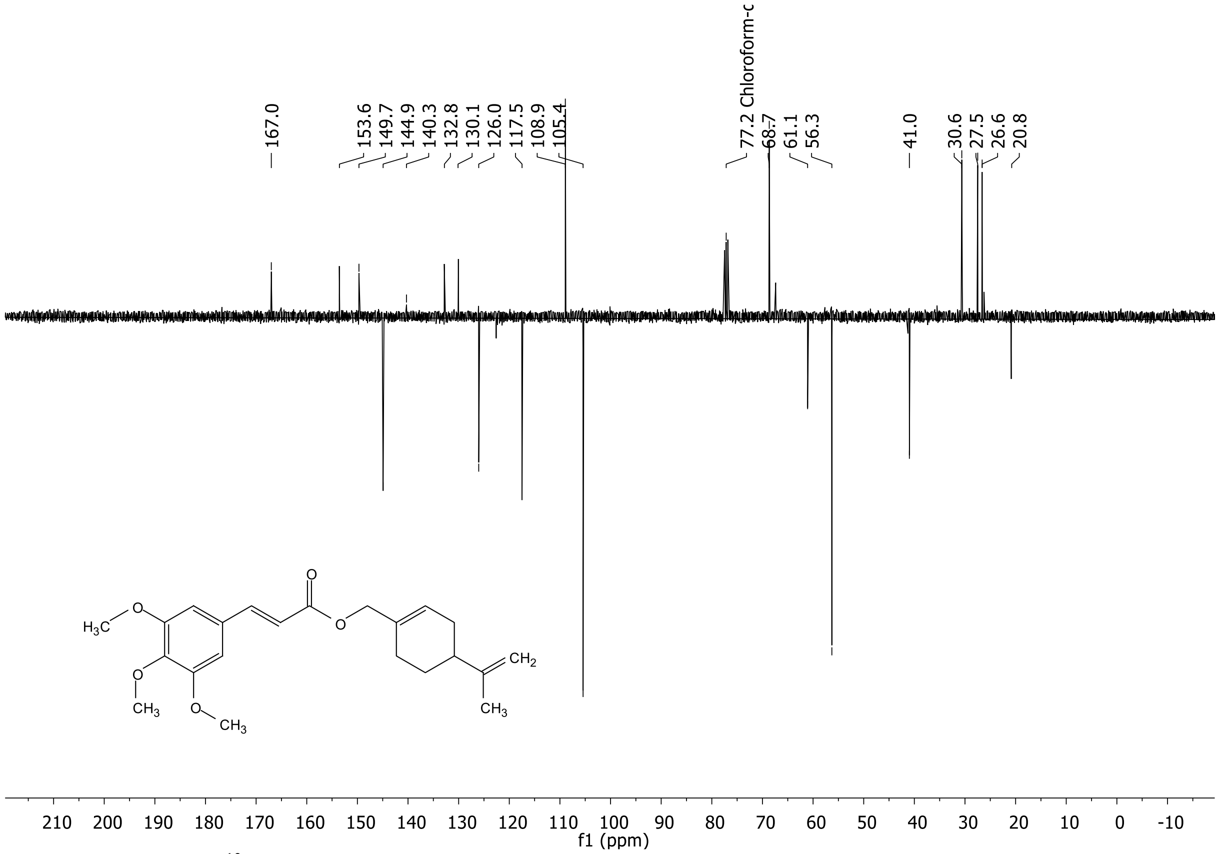

Figure S38: APT-^{13}C NMR spectrum (100 MHz, $CDCl_3$) of (*S*,*E*)-(4-(prop-1-en-2-yl)cyclohex-1-en-1-yl)methyl 3-(3,4,5-trimethoxyphenyl)acrylate (**12**).

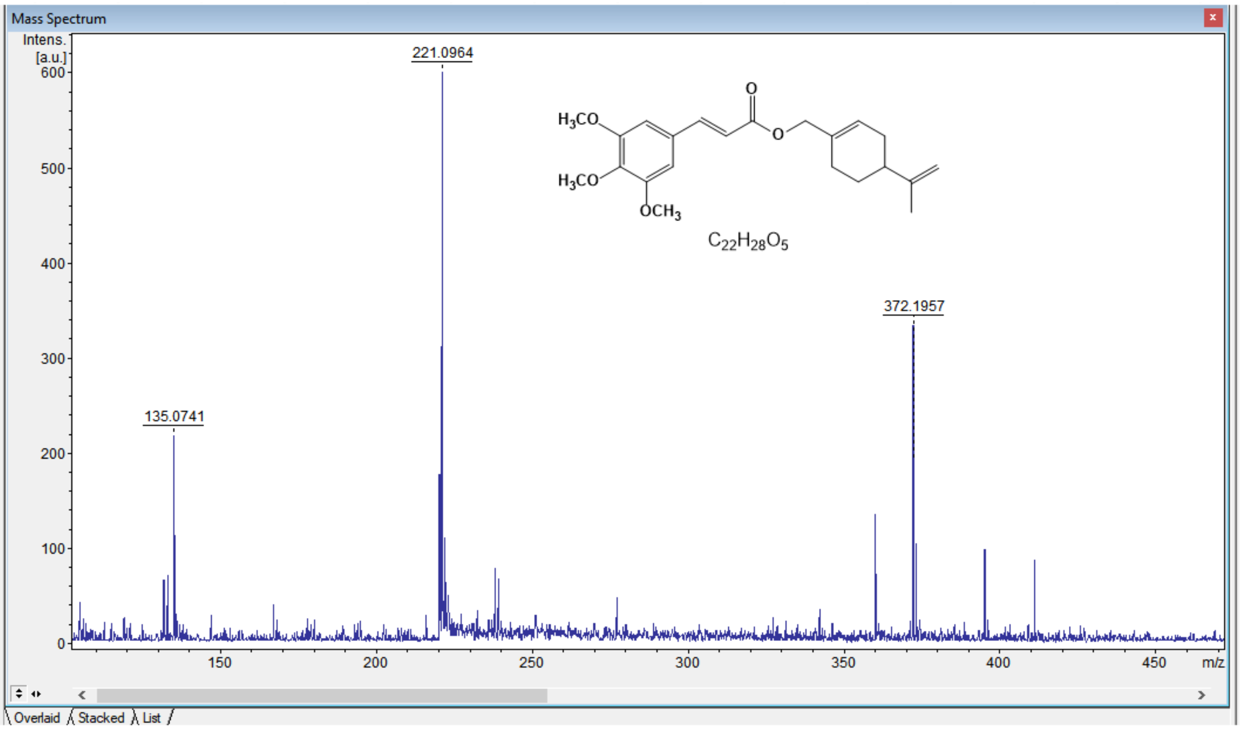

Figure S39: HRMS spectrum (MALDI TOF/TOF) of (*S*,*E*)-(4-(prop-1-en-2-yl)cyclohex-1-en-1-yl)methyl 3-(3,4,5-trimethoxyphenyl)acrylate (**12**).

((*E*)-(*1S,2R,4S*)-1,7,7-trimethylbicyclo[2.2.1]heptan-2-yl 3-(3,4,5-trimethoxyphenyl)acrylate (**13**): colorless oil; Yield: 46.2%; Reaction time: 24 h; TLC (7:3 Hex/EtOAc), R_f = 0.70; IR *ν*max (KBr, cm^{-1}): 2956, 2878, 1709, 1638, 1584, 1508, 1459, 1276, 1129, 828; **^{1}H NMR ($CDCl_3$, 400 MHz):** δH 7.59 (*d*; *J*=15.90 Hz; 1H), 6.76 (*s*; 2H), 6.36 (*d; J*= 15.90 Hz; 1H), 5.02 (*ddd*; *J*= 9.9; 3.4; 2.1 Hz; 1H), 3.88 (*s*, 9H), 2.48 – 2.37 (*m*; 1H), 2.10 – 2.01 (*m*; 1H), 1.81 – 1.74 (*m;* 1H), 1.71 (*t; J*= 4.5 Hz; 1H), 1.40 – 1.32 (*m;* 1H), 1.30 – 1.25 (*m;* 1H), 1.05 (*dd; J*= 16.1; 6.5 Hz; 1H), 0.95 (*s*; 3H), 0.90 (*s*; 3H), 0.88 (*s;* 3H); **^{13}C NMR ($CDCl_3$, 100 MHz):** δC 167.4, 153.6, 144.3, 140.2, 130.2, 118.2, 105.4, 80.2, 61.1, 56.3, 49.1, 48.0, 45.1, 37.0, 28.2, 27.4, 19.9, 19.0, 13.7 [7].

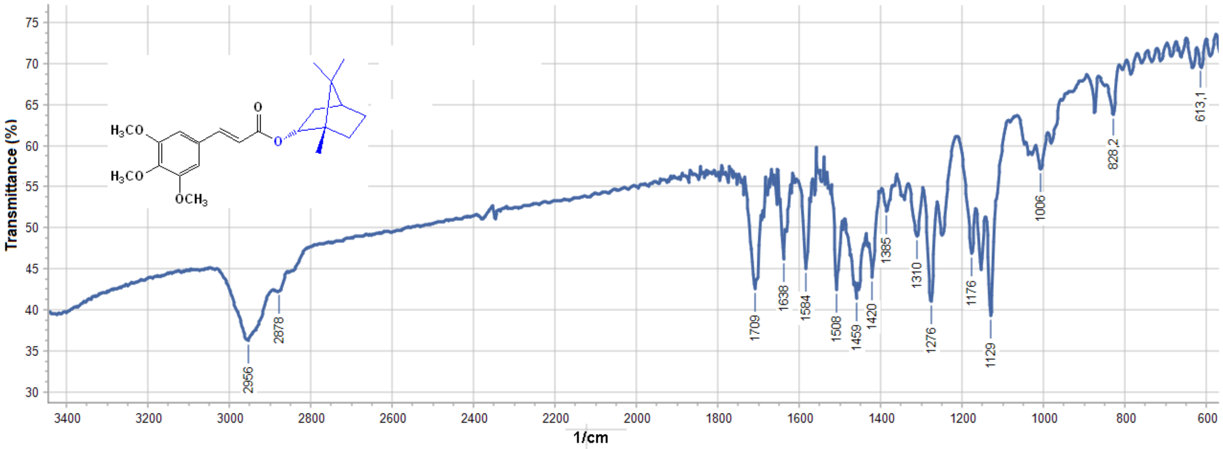

Figure S40: Infrared spectrum (KBr, cm^{-1}) of ((*E*)-(*1S,2R,4S*)-1,7,7-trimethylbicyclo[2.2.1]heptan-2-yl 3-(3,4,5-trimethoxyphenyl)acrylate (**13**).

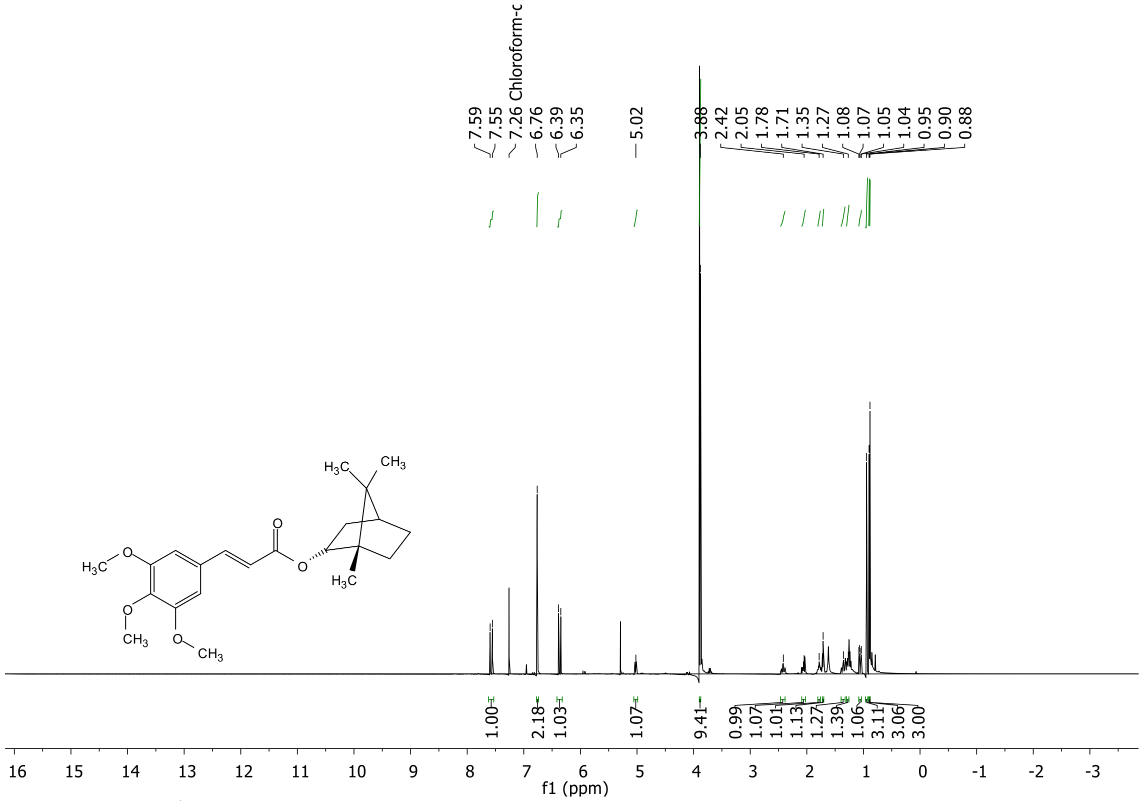

Figure S41: ^{1}H NMR spectrum (400 MHz, $CDCl_3$) of ((*E*)-(*1S,2R,4S*)-1,7,7-trimethylbicyclo[2.2.1]heptan-2-yl 3-(3,4,5-trimethoxyphenyl)acrylate (**13**).

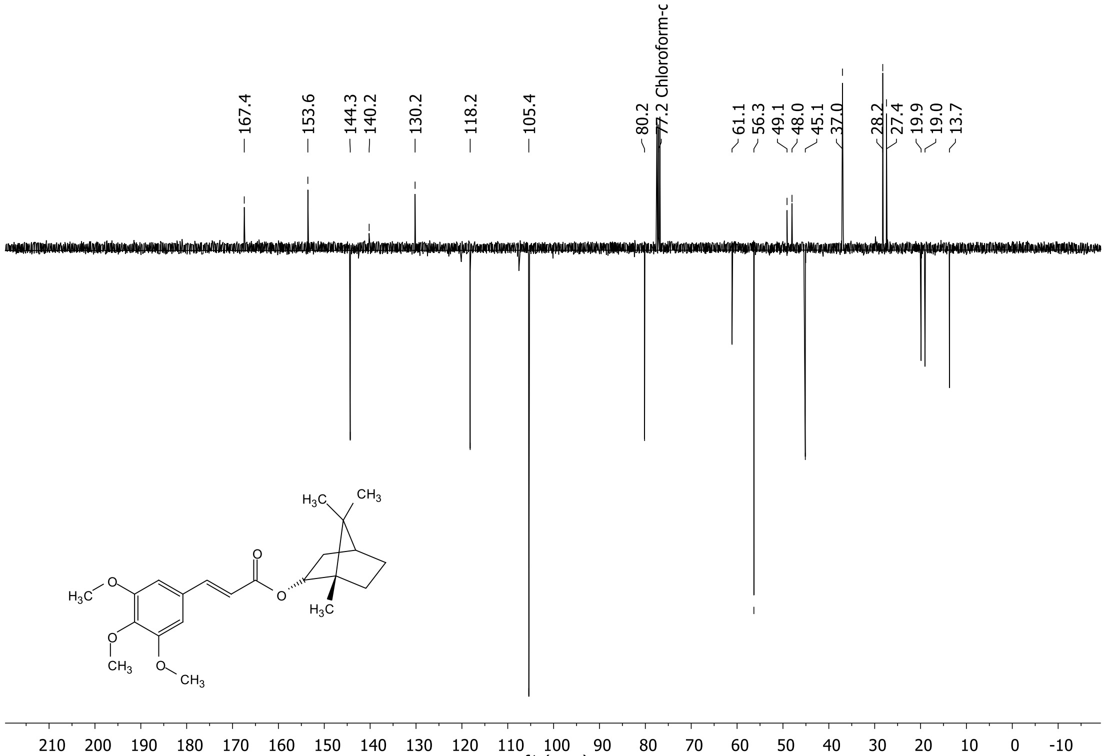

Figure S42: APT-^{13}C NMR (100 MHz, $CDCl_3$) of ((*E*)-(*1S,2R,4S*)-1,7,7-trimethylbicyclo[2.2.1]heptan-2-yl 3-(3,4,5-trimethoxyphenyl)acrylate (**13**).

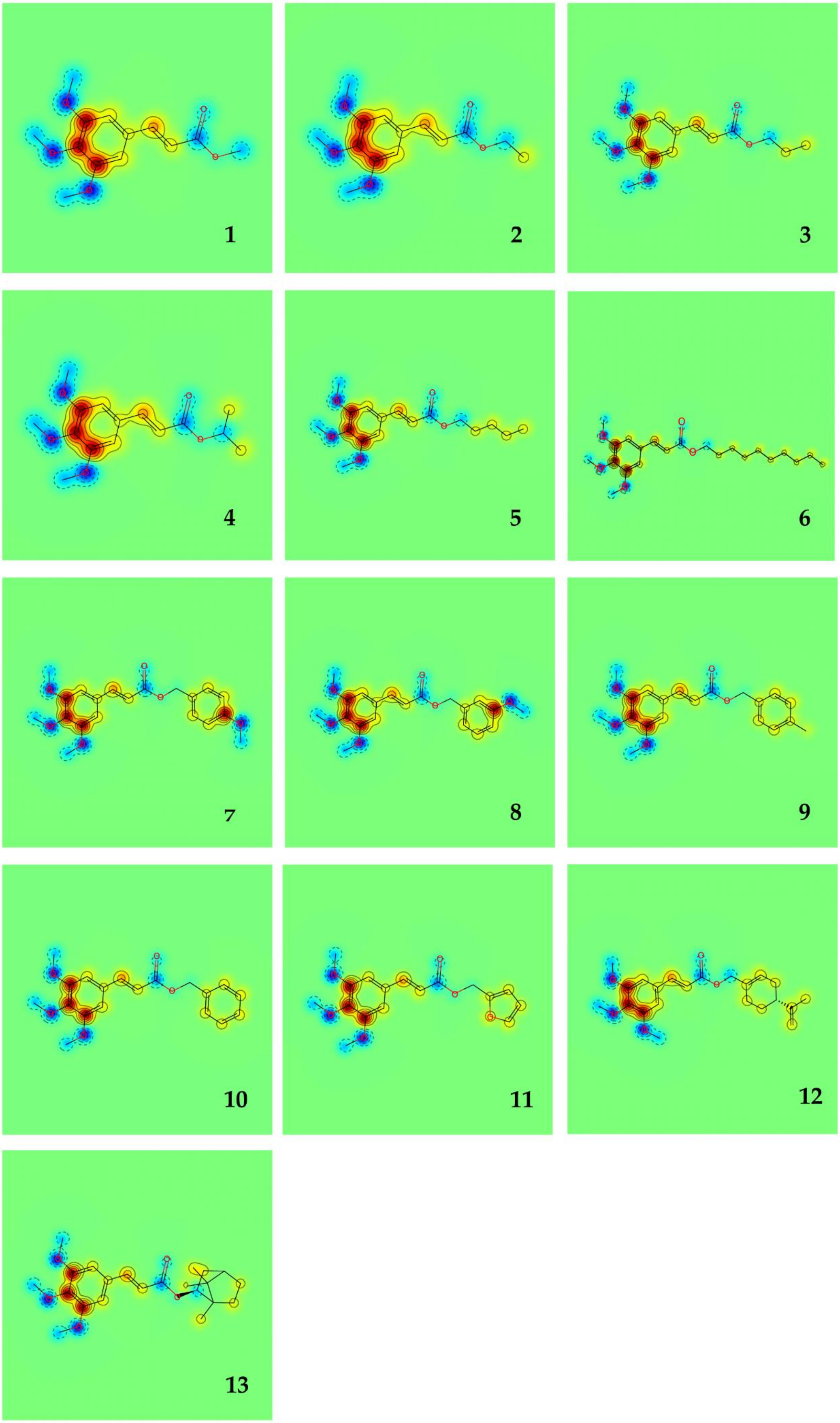

Figure S43: Atomic contributions to logP for compounds **1-13**. The color scale goes from blue (hydrophilic) to red (hydrophobic).

Table S1. Predicted logarithm of the partition coefficients for compounds **1-13.**

Compound	logP
**1**	1.8986
**2**	2.2887
**3**	2.6788
**4**	2.6772
**5**	3.459
**6**	5.4095
**7**	3.4776
**8**	3.4776
**9**	3.77742
**10**	3.469
**11**	3.062
**12**	4.5714
**13**	4.4836

References

[1] Kumar, S.; Arya, P.; Mukherjee, C.; Singh, B. K.; Singh, N.; Parmar, V. S.; Prasad, A. K.; Ghosh, B. Novel Aromatic Ester from *Piper Longum* and Its Analogues Inhibit Expression of Cell Adhesion Molecules on Endothelial Cells. *Biochemistry* **2005**, *44* (48), 15944–15952. https://doi.org/10.1021/bi050941u.

[2] Jung, J.-C.; Moon, S.; Min, D.; Park, W. K.; Jung, M.; Oh, S. Synthesis and Evaluation of a Series of 3,4,5-Trimethoxycinnamic Acid Derivatives as Potential Antinarcotic Agents. *Chem Biol Drug Des* **2013**, *81* (3), 389–398. https://doi.org/10.1111/cbdd.12087.

[3] da Nóbrega, F.; Ozdemir, O.; Nascimento Sousa, S.; Barboza, J.; Turkez, H.; de Sousa, D. Piplartine Analogues and Cytotoxic Evaluation against Glioblastoma. *Molecules* **2018**, *23* (6), 1382. https://doi.org/10.3390/molecules23061382.

[4] Zhao, Z.; Bai, Y.; Xie, J.; Chen, X.; He, X.; Sun, Y.; Bai, Y.; Zhang, Y.; Wu, S.; Zheng, X. Excavating Precursors from the Traditional Chinese Herb Polygala Tenuifolia and Gastrodia Elata: Synthesis, Anticonvulsant Activity Evaluation of 3,4,5-Trimethoxycinnamic Acid (TMCA) Ester Derivatives. *Bioorg Chem* **2019**, *88*, 102832. https://doi.org/10.1016/j.bioorg.2019.03.006.

[5] Katritzky, A. R.; Zhang, S.; Soares, A.; Wang, M. A Facile Synthesis of Benzyl-α, β-Unsaturated Carboxylic Esters. *ARKIVOC* **2001**, *10*, 54–57.

[6] Nóbrega, F. R.; Silva, L. V.; Bezerra Filho, C. D. S. M.; Lima, T. C.; Castillo, Y. P.; Bezerra, D. P.; Sousa, D. P. D. Design, antileishmanial activity, and QSAR studies of a series of piplartine analogues. *J. Chem.*, ***2019***, 2019.

[7] Turkez, H.; Nóbrega, F. R. da; Ozdemir, O.; Bezerra Filho, C. da S. M.; Almeida, R. N. de; Tejera, E.; Perez-Castillo, Y.; Sousa, D. P. de. NFBTA: A Potent Cytotoxic Agent against Glioblastoma. *Molecules* **2019**, *24* (13), 2411. https://doi.org/10.3390/molecules24132411.
